# *Vaccinium vitis-idaea* L. Fruits: Chromatographic Analysis of Seasonal and Geographical Variation in Bioactive Compounds

**DOI:** 10.3390/foods10102243

**Published:** 2021-09-22

**Authors:** Gabriele Vilkickyte, Lina Raudone

**Affiliations:** 1 Laboratory of Biopharmaceutical Research, Institute of Pharmaceutical Technologies, Lithuanian University of Health Sciences, Sukileliu Avenue 13, LT-50162 Kaunas, Lithuania; lina.raudone@lsmuni.lt; 2 Department of Pharmacognosy, Lithuanian University of Health Sciences, Sukileliu Avenue 13, LT-50162 Kaunas, Lithuania

**Keywords:** *Vaccinium*, lingonberry, phenolics, triterpenoids, phenology, chemodiversity, liquid chromatography

## Abstract

*Vaccinium vitis-idaea* L. (lingonberry) fruits are promising sources of bioactive components with high potential in biomedical applications. Selection in plant breeding, determination of perspective wild clones with optimal growing conditions, and appropriate harvesting time leading to standardized extracts are key factors for achieving phytochemical quality to meet consumer’s needs. In the present study, lingonberry fruits collected along different phenological stages and from different geographical locations were analyzed for the composition of 56 constituents using validated chromatographic techniques. Early stages of lingonberries vegetation were determined as the best stages for obtaining high levels of most phenolics and triterpenoids, while the end of berry vegetation could be chosen as the optimal harvesting time in terms of anthocyanins. Furthermore, intensified continuous biosynthesis of triterpenoids and phenolic acids precursors after vegetation season in the winter sample was observed. Chemodiversity of lingonberries was affected by geographical factors as well as climatic and edaphic conditions, indicating different favorable growing conditions for the accumulation of particular compounds. Present findings could serve for breeders to obtain the highest yields of desirable lingonberry constituents, relevant in food and pharmaceutical industries.

## 1. Introduction

The phytochemical composition, quality, and safety of plant-based food are typically influenced by numerous factors [1]. Plants are exposed to substantial phenological variations, including environmental and seasonal fluctuations over the course of the year [2]. Although the metabolic processes leading to the accumulation of bioactive constituents are basically controlled by genetic makeup, physiological conditions and harvesting time are key essential factors that influence bioactive compounds contents in different organs of medicinal plants [3,4,5]. Great variability in phytochemical composition and quantity could also be attributed to plant geographical origin and edaphic factors [6]. Plants have cells known as circadian clock genes, and thus are able to anticipate seasonal changes and adapt to different growing conditions by regulating the development and accumulation of bioactive compounds in the presence of any external or environmental cues [7]. Relations among the accumulation and storage of secondary plant metabolites due to abiotic stresses have not yet been fully defined, and effects are likely to vary depending on the plant, organ, and type of metabolites [3]. Therefore, it is of great importance from the plant material quality point of view to study relations of growth conditions and chemical composition of edible plants.

*Vaccinium vitis-idaea* L. (lingonberry) belonging to the family *Ericaceae* Juss. and the genus *Vaccinium* L. bear an annual crop of sour-tart taste reddish color berries which has been widely used in traditional Scandinavian diets and are also widespread in Baltic States, Russia, and North America [8,9]. Lingonberry fruits are regarded as nonpoisonous, natural, and safe food, which can be consumed not only in fresh and frozen forms but also processed into juice, wine, jam, jellies, pastries, and other desserts [10,11]. These products are often classified as ‘superfoods’ due to their richness of dietary micronutrients and bioactive compounds [9]. Lingonberries contain a significantly higher anthocyanin level than other commonly consumed berries [12]. The presence of a wide variety of phenolic and triterpenic compounds in lingonberries was reported previously [13,14,15,16,17]. The high content of these constituents is thought to be linked to antioxidant, anti-inflammatory, antiproliferative, antimicrobial, antiobesity, and hepatoprotective properties of lingonberries. Therefore, they are increasingly marketed as a natural solution for the urinary tract and digestive health [18,19,20]. Lingonberry components are regarded as promising lead compounds for multi-targeting pharmaceuticals and nutraceuticals of natural origin [21]. However, data concerning the influence of phenological stage and geographical conditions on the accumulation of secondary metabolites are still scarce [22].

To the best of our knowledge, full-spectrum analysis of individual bioactive components in correlation to the harvesting date, environmental conditions, geographical gradients, and soil quality parameters has never been reported in wild-growing lingonberry fruits before. Since the selection of optimal phenological-growing stage ensures the highest loadings of required bioactive compounds [23], it can be an important point for quality assurance of lingonberry’s therapeutic properties. Co-variation in the production of defensive metabolites in response to various stresses and possible compositional changes in phenolics and triterpenoids during vegetation would have a scientific value in terms of the metabolic pathways and decay processes of these compounds [24]. Whereas, geographical and edaphic factors analysis in a relation to phytochemical profiles may help to identify different chemophenotypes and highlight favorable growing conditions [25] for possible lingonberry uses in food and pharmaceutical industries.

Given these considerations, our research aimed at investigating the effects of phenological and geographical conditions, as well as environmental, edaphic, and internal factors on phenolics and triterpenoids accumulation in wild-growing lingonberry fruits using validated UPLC (ultra performance liquid chromatography)- and HPLC-PDA (high performance liquid chromatography-photodiode array detection) methods.

## 2. Materials and Methods

### 2.1. Plant Material and Collecting Conditions

A study of phenological variance was carried out during one vegetative season in 2019 at Apūniškis forest, Lithuania (latitude: 56.01°; longitude 25.52°; altitude: 110 m; forest type—*Pinetum vaccinio-myrtilosum*). Samples of lingonberry fruits were collected from 10 randomly selected shrubs at the same forest plots on 1, 15, and 29 July (berry formation stages); 11 and 24 August (berry ripening); 6 and 20 September (massive berry ripening); 4 October (end of the vegetation); and 28 December (non-vegetative stage). Collecting dates were chosen according to the lingonberry vegetative stages characterization in Lithuania [26]. Climatic data of average monthly temperature (°C), precipitation (mm), humidity (%), and sunshine duration (h) (Supplementary Figure S1) were provided by the Lithuanian Hydrometeorological Service under the Ministry of Environment of the Republic of Lithuania.

**Figure 1:**
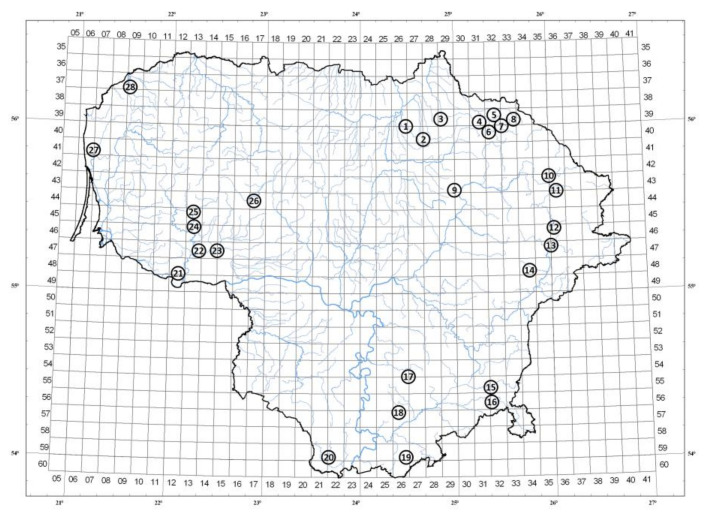
Collecting locations of lingonberry samples in Lithuania. Assignments are indicated in Table 1.

Analysis of phytogeographical variance was performed at the end of September 2019. Lingonberry fruits were collected from different regions of Lithuania in pine, spruce, or birch tree forests with different edaphic conditions (Figure 1, Table 1) from 10 shrubs in each habitat, applying randomized experimental design. Sample of Labanoras location differed by the variety: (a) *V. vitis-idaea* L. var. *leucocarpum* Asch. et Magnus, which distinguished themselves by white berries; and (b) typical variety of lingonberries, growing together in certain forest plots. Soil samples in the plant root zones were taken in each collecting location and were subjected to pH value, contents of nitrogen (N), potassium (K), phosphorus (P), calcium (Ca), magnesium (Mg), chloride (Cl), and electrical conductivity (EC) determination in Agrochemical Research Laboratory (Lithuanian Research Centre for Agriculture and Forestry). To measure P and K concentrations, soil samples were extracted using 0.5 M of acetic acid/sodium acetate buffer (1:10, *w*/*v*) with pH 3.4–3.6; contents of N, Ca, Mg, Cl, and EC were determined in a soil–water extract (1:10, *w*/*v*), while pH was measured in 1M of KCl extract (1:5, *w*/*v*). The FIAstar 5000 Analyzer (Foss Analytical A/S, Denmark) was used and potentiometric and colorimetric methods were applied (ISO 14256:2005, ISO 10390:2005).

Collected lingonberries were immediately frozen and lyophilized in a ZIRBUS sublimator 3 × 4 × 5/20 (Bad Grund, Germany) at a pressure of 0.01 mbar and condenser temperature of –85 °C. Freeze-dried fruits were ground with a Retsch 200 mill (Haan, Germany) to homogenous powder and kept in sealed containers. Before extraction, residual moisture was determined by moisture analyzer Presica 310M (Dietikon, Switzerland) and all obtained results were re-calculated for dried weight (DW) raw material.

### 2.2. Chemicals and Solvents

Analytical and chromatographic grade solvents were used for this study: acetonitrile, acetone, and methanol from Sigma-Aldrich (Steinheim, Germany); and trifluoroacetic and formic acids from Merck (Darmstadt, Germany). Ultrapure water used in this study was Milli-Q quality (Millipore, Bedford, MA, USA). The following high-purity standards were used: procyanidins B1, B2, C1, A1, A2, A4, (+)-catechin, (−)-epicatechin, quercetin, quercitrin (quercetin-3-*O*-rhamnoside), isoquercitrin (quercetin-3-*O*-glucoside), avicularin (quercetin-3-*O*-arabinofuranoside), guaiaverin (quercetin-3-*O*-arabinopyranoside), rutin (quercetin-3-*O*-rutinose), reynoutrin (quercetin-3-*O*-xyloside), kaempferol, afzelin (kaempferol-3-*O*-rhamnoside), astragalin (kaempferol-3-*O*-glucoside), *trans*-cinnamic, caffeic, chlorogenic (3-*O*-caffeoylquinic), neochlorogenic (5-*O*-caffeoylquinic), cryptochlorogenic (4-*O*-caffeoylquinic), *p*-coumaric, ferulic, sinapic, benzoic, vanillic, and protocatechuic acids, arbutin, resveratrol, α-amyrin, β-amyrin, β-sitosterol, lupeol, erythrodiol, maslinic and oleanolic acids from Sigma-Aldrich; ursolic acid from Carl Roth (Karlsruhe, Germany); delphinidin-3-*O*-glucoside, delphinidin-3-*O*-galactoside, cyanidin-3-*O*-galactoside, cyanidin-3-*O*-glucoside, cyanidin-3-*O*-arabinoside, petunidin-3-*O*-glucoside, peonidin-3-*O*-glucoside, malvidin-3-*O*-glucoside, cyanidin chloride, procyanidin B3, hyperoside (quercetin-3-*O*-galactoside), uvaol, friedelin, betulin, and betulinic and corosolic acids from Extrasynthese (Genay, France).

### 2.3. Extraction and Chromatographic Analysis

#### 2.3.1. Estimation of Anthocyanins

In order to extract anthocyanins, freeze-dried powdered lingonberry samples (1.0 g) were extracted three times with 10 mL of acidified methanol in an Elmasonic P 120H ultrasonic bath (Singen, Germany) for 15 min (Frequency, 80 kHz; power intensity, 100% (330 W)). Supernatants of each extraction were pooled after centrifugation for 10 min at 3000× *g* in Biofuge Stratos centrifuge (Hanau, Germany).

Anthocyanins were analyzed by direct injection of extracts, previously filtered through PVDF membrane filters with a pore size of 0.22 μm (Carl Roth GmbH, Karlsruhe, Germany). Chromatographic separation was performed using Waters ACQUITY UPLC system (Water, Milford, MA, USA) equipped with a PDA detector and an ACE Super C18 (100 × 2.1 mm, 1.7 μm) column (ACT, Aberdeen, UK), operated at a constant temperature of 30 °C. The gradient elution program consisting of 10% (*v*/*v*) formic acid (A) and 100% (*v*/*v*) acetonitrile (B) was as follows: 0 min, 5% B; 0–2 min, 5–9% B; 2–7 min, 9–12% B; 7–9 min, 12–25% B; 9–10 min, 25–80% B; 10–10.5 min, 80% B; 10.5–11 min, 80–5% B; and 11.0–12.0 min, 5% B. The flow rate was 0.5 mL/min, and the injection volume was 1 μL. All anthocyanins were identified and quantified at 520 nm wavelength based on a comparison of retention times with those of standards. For quantification, calibration curves were constructed by plotting the response of each analyte versus concentration. Validation data and representative chromatograms could be found in our previous paper [27].

#### 2.3.2. Estimation of Other Phenolics

For the analysis of other phenolic compounds (proanthocyanidins, flavan-3-ols, flavonols, phenolic acids, their precursors, arbutin derivatives, and stilbenes), about 0.4 g of fruits powder was weighted and 10 mL of 70% *v*/*v* acetone was added. Ultrasonic extraction continued for 15 min with centrifugation for 30 min at 3000× *g* and filtration via 0.22 μm PVDF membrane filters afterward.

Phenolics were analyzed using Waters e2695 Alliance HPLC system (Waters, Milford, MA, USA) equipped with a 2996 PDA detector. An ACE Super C18 (250 mm × 4.6 mm, 3 μm) column (ACT, Aberdeen, UK), maintained at 35 °C, was used. Sample volumes of 10 μL were injected and separation was achieved using the following gradient: 0.1% (*v*/*v*) trifluoroacetic acid (A) and acetonitrile (B) with gradient elution formed as follows: 0 min, 10% B; 0–40 min, 30% B; 40–60 min, 70% B; 60–64 min, 90% B; 64–70 min, 10% B at the flow rate of 0.5 mL/min. Detection was performed by scanning from 220 to 380 nm and quantification according to 5–7 points linear calibration curves of external standards. Only well-known lingonberry constituents, i.e., 2-*O*-caffeoylarbutin and quercetin-3-*O*-(4″-(3-hydroxy-3-methylglutaryl)-rhamnoside (quercetin-HMG-rhamnoside), due to commercial unavailability, were tentatively quantified using calibration curves of arbutin and quercetin, respectively. Their identity was previously confirmed by mass spectrometry. HPLC-PDA method validation parameters with chromatograms were presented elsewhere [28].

#### 2.3.3. Estimation of Triterpenoids

Ultrasound-assisted extraction of triterpenoids was achieved by weighting approx. 1.0 g of each sample and extracting with 10 mL of methanol for 25 min. The extracts were then centrifuged for 30 min at 3000× *g* and filtered through 0.22-μm PVDF membrane filters to the dark glass vials.

HPLC-PDA separation of triterpenoids was carried out using an ACE C18 (150 × 4.6 mm, 3 µm) column (ACT, Aberdeen, UK). Maslinic, corosolic, betulinic, oleanolic, ursolic acids, betulin, erythrodiol, and uvaol were analyzed using acetonitrile and water as the mobile phase components in the isocratic mode (89:11, *v*/*v*) at the flow rate of 0.7 mL/min and column temperature maintained at 20 °C. Lupeol, β-amyrin, α-amyrin, friedelin, and β-sitosterol were determined using the mobile phase consisted of acetonitrile and methanol (10:90, *v*/*v*), delivered at a flow rate of 0.7 mL/min in the isocratic mode when column temperature was set at 35 °C. All triterpenoids were injected at the volume of 10 µL. Identification was performed at a wavelength of 205 nm by comparing retention times to those of standard compounds and linear regression models were obtained for quantification purposes. Validation data of chromatographic methods presented above are given in our previous report [29].

### 2.4. Statistical Analysis

All data were reported as mean ± standard deviation (SD) of at least three experiments. Significant differences between means were determined by one-way ANOVA, followed by Tukey HSD multiple comparison test. Pearson’s correlation coefficients were calculated to assess associations between different parameters. The quantitative analysis data were further analyzed by principal component analysis and hierarchical cluster analysis using the between groups clustering method with squared Euclidean distances. Results were processed using IBM SPSS Statistics version 26.0 (SPSS Inc., Chicago, IL, USA) package and Microsoft Office Excel 2016 software (Microsoft, Redmond, WA, USA), applying a significance level of α = 0.05.

## 3. Results

### 3.1. Seasonal Patterns of Secondary Metabolites Accumulation in Lingonberry Fruits

**Figure 2:**
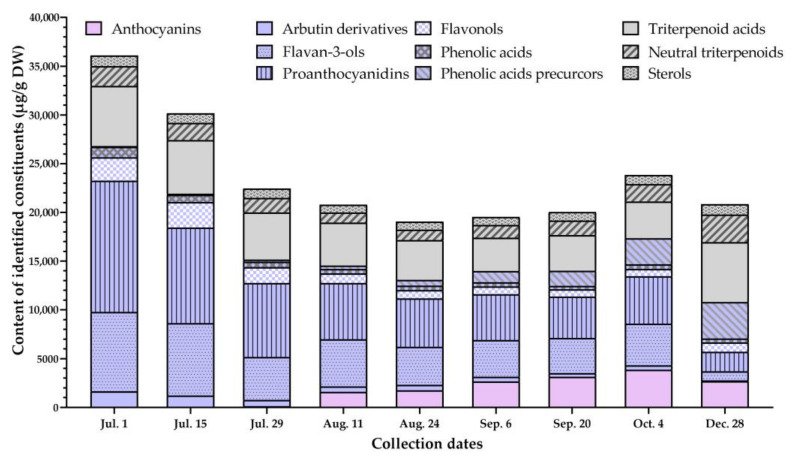
Variation of phenolics and triterpenoids in lingonberries (μg/g DW) during different phenological stages.

A total of 56 bioactive compounds, belonging to groups of phenolics and triterpenoids, were identified and quantified during different growth stages in the samples of lingonberry fruits. The highest level of total identified constituents was reached at the early stages of berry vegetation; during ripening, this level decreased and slightly re-increased at the end of the vegetation (total contents of different subgroups of phenolics and triterpenoids are depicted in Figure 2).

#### 3.1.1. Phenological Analysis of Phenolics

The most abundant group of the analyzed constituents was phenolics, the content of which in the early growth stages (1 July) made up 74.3% of total constituents; with berry ripening their level reduced by 1.5-fold (20 September), then slightly re-increased at the end of the vegetation (4 October) and further decreased due to decay (28 December) (Table 2). The significant variance of contents of particular lingonberry phenolics was observed with the greatest variation of anthocyanins (coefficient of variation (CV) = 82.5%).

Anthocyanins comprised less than 1.0% of total identified bioactive compounds in unripe fruits (samples from July), while the highest (*p* < 0.05) contribution and contents were recorded in the samples collected at the end of the vegetation—4 October (16.2%; 3862.0 μg/g DW, respectively). This level decreased by 30.6% on 28 December. Anthocyanins profile consisted mainly of cyanidin-3-*O*-galactoside, cyanidin-3-*O*-glucoside, and cyanidin-3-*O*-arabinoside, which accounted for 97.5–100.0% of total identified anthocyanins in different samples. Minor lingonberry anthocyanins included delphinidin-3-*O*-galactoside, delphinidin-3-*O*-glucoside, petunidin-3-*O*-glucoside, peonidin-3-*O*-glucoside, malvidin-3-*O*-glucoside, and cyanidin aglycone, which were not detected in unripe lingonberries at all. Cyanidin-3-*O*-galactoside was the only anthocyanin marker that was found during all phenological stages. The total content of anthocyanins was negatively correlated with precipitation, but positively with air humidity (*r* = –0.722 and 0.712, respectively, *p* < 0.05).

Proanthocyanidins (procyanidins A1, A2, A4, B1, B2, B3, C1) accounted up to 50.2% for identified phenolics and were considered to be the largest subgroup in all vegetative stages with maximum (*p* < 0.05) contents at the beginning of the vegetation (total amount reached 13,477.3 μg/g DW) and notably reduced level in a sample collected in winter (2015.2 μg/g DW). Procyanidin B3 was the predominant proanthocyanidin in samples of 1 July–11 August, while other samples were distinguished by significantly highest levels of procyanidin B1. The contents of proanthocyanidins correlated negatively (*r* = −0.800, *p* < 0.05) with chemically related compounds, i.e., anthocyanins, and positively (*r* = 0.951, *p* < 0.05) with flavan-3-ols. Prevailing (+)-catechin and (−)-epicatechin comprised a group of flavan-3-ols and the sum of them was in a range of 926.1–8139.4 μg/g DW, contributing up to 33.8% of identified phenolics, with the highest (*p* < 0.05) content found on 1 July and the lowest (*p* < 0.05) on 28 December. Amounts of flavan-3-ols and proanthocyanidins highly correlated with air temperature, precipitation, sunshine duration, and negatively with air humidity (*r* up to 0.847, 0.981, 0.801, and −0.724, respectively, *p* < 0.05).

Flavonols, namely flavonol aglycones (kaempferol, quercetin), kaempferol glycosides (astragalin, afzelin), and quercetin glycosides (quercitrin, quercetin-HMG-rhamnoside, rutin, hyperoside, isoquercitrin, reynoutrin, guaiaverin, avicularin), which made up to 8.7% of total identified constituents and up to 12.0% of identified phenolics, was the fourth large subgroup. In early vegetative stages (1–15 July), isoquercitrin was the prevailing flavonol, later surpassed by quercitrin. The greatest content of total identified flavonols (2626.0 μg/g DW) in fruit samples collected on 15 July was found. This level reduced by 3.6-fold on 4 October and then re-increased (*p* < 0.05) till 962.1 μg/g DW on 28 December. A similar phenological pattern was found of phenolic acids, the total content of which was highly correlated with the level of flavonols (*r* = 0.927, *p* < 0.05).

Phenolic acids accounted for 1.8–3.0% of total identified constituents and up to 4.0% of identified phenolics. This complex was comprised of hydroxycinnamic (chlorogenic, cryptochlorogenic, neochlorogenic acid, *p*-coumaric, sinapic, ferulic) and hydroxybenzoic (vanillic and protocatechuic) acids, with chlorogenic acid regarded as main in all samples. The contents of phenolic acids were relatively similar throughout different phenological stages with CV (40.7%) being the lowest out of the phenolics group. The greatest accumulation of phenolic acids was observed at the earliest stage of berry formation (1066.3 μg/g DW), which was reduced up to 359.1 μg/g DW on 20 September and re-increased to 497.5 μg/g DW afterward. A negative moderate correlation (*r* = −0.650, *p* < 0.05) was found between contents of phenolic acids and phenolic acids precursors (*trans*-cinnamic and benzoic acid) in samples of different phenological stages.

Phenolic acids precursors contributed up to 17.9% of total identified metabolites (0.4–34.6% of total phenolics) with the lowest (*p* < 0.05) contents found in samples collected on 1 July (total amount reached 101.0 μg/g DW), consistently increasing till 28 December (3738.4 μg/g DW). The total content of phenolic acids precursors highly negatively correlated with air temperature, precipitation, sunshine duration, and positively with air humidity (*r* = −0.982, −0.850, −0.992, and 0.977, respectively, *p* < 0.05). Furthermore, the content of benzoic and *trans*-cinnamic acids was negatively correlated with the level of arbutin derivatives (*r* = −0.823, *p* < 0.05), showing different accumulation patterns.

Contents of arbutin and 2-*O*-caffeoylarbutin were the highest (*p* < 0.05) in samples collected on 1 July—1518.7 and 128.7 μg/g DW, while winter samples were distinguished by 22- and 9-fold lower levels, respectively. Arbutin was one of the main phenolics in unripe fruits, accounting for 4.2% of the total identified constituents. Resveratrol, belonging to the subgroup of stilbenes, was regarded as a minor lingonberry compound at a quantifiable level (0.5 μg/g DW), and was detected only in samples collected at the earliest stage of berry formation.

#### 3.1.2. Phenological Analysis of Triterpenoids

Another group of identified secondary metabolites was triterpenoids, which accounted for 25.7–48.3% of total identified compounds with the highest (*p* < 0.05) contributions detected in samples collected in winter (Table 3). The accumulation pattern of initial high level in unripe fruits, decreasing during berry ripening and re-increasing at the end of the vegetation was found in case of most triterpenoids. Triterpenoid acids (maslinic, corosolic, betulinic, oleanolic, and ursolic) were the most abundant subgroup of triterpenoids (accounted for 58.1–70.6%). The highest (*p* < 0.05) contents of triterpenoid acids were recorded in samples collected on 1 July and 28 December (total amounts reached 6162.4 and 6143.9 μg/g DW, respectively), while a sample of 6 September was distinguished by the lowest (*p* < 0.05) level (3411.2 μg/g DW). Different ratios (1:4.4–1:5.8) of oleanolic and ursolic acids were found in samples of different collecting dates, with ursolic acid being predominant, contributing up to 23.6% of total identified lingonberry constituents and up to 58.9% of total triterpenoids.

Neutral triterpenoids, namely betulin, erythrodiol, uvaol, lupeol, α-amyrin, β-amyrin, and friedelin, accounted for 4.9–13.5% of total identified constituents and up to 28.0% of total triterpenoids, so was regarded as the second large subgroup. Sum contents varied between 1016.9 μg/g DW in a sample of 11 August and 2824.6 μg/g DW of 28 December. α-Amyrin was the main neutral triterpenoid, which prevailed over others in samples of all phenological stages. Strong negative correlations were found between the sum content of neutral triterpenoids to air temperature and sunshine duration (*r* = −0.878, −0.822, respectively, *p* < 0.05). Amounts of neutral triterpenoids increased in a similar manner as contents of β-sitosterol with a positive correlation among them (*r* = 0.942, *p* < 0.05).

Amounts of β-sitosterol, belonging to the subgroup of sterols, did not vary largely with phenological stages (CV = 11.6%). This compound contributed up to 5.3% of total identified constituents and up to 15.0% of total triterpenoids with the greatest (*p* < 0.05) contents determined in samples of 1 July and 28 December, on average 1096.7 μg/g DW. Whereas, approx. 1.3-fold lower accumulation was found during the vegetation on 11 August.

#### 3.1.3. Hierarchical Cluster Analysis of Phenological Data

**Figure 3:**
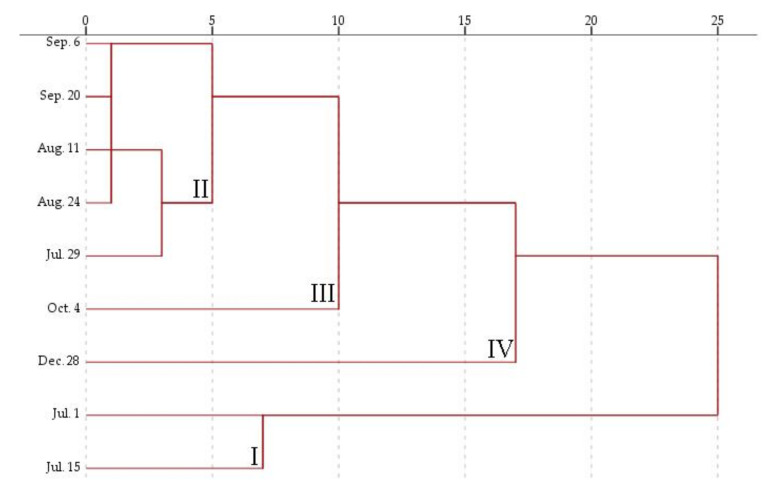
Dendrogram estimating contents of phenolics and triterpenoids in lingonberries based on their collection date. Assignments I-IV indicate cluster number.

Phenolics and triterpenoids composition data were subjected to hierarchical cluster analysis, based on different phenophases (Figure 3). Lingonberry fruits were grouped into four statistically significant clusters (*p* < 0.05). The first cluster comprised unripe lingonberry samples, collected in the first half of June. These samples were distinguished by the highest contents of most phenolics belonging to subgroups of flavan-3-ols, proanthocyanidins, flavonols, phenolic acids, arbutin derivatives, stilbenes, higher than average contents of triterpenoids, but the lowest only traceable amounts of anthocyanins and low contents of benzoic and *trans*-cinnamic acids. Samples of intensive berry formation and ripening stages (29 July–20 September) formed the second cluster. These samples differed from the others by the lowest contents of most neutral triterpenoids, sterols, and triterpenoid acids, and average contents of phenolics. The third cluster was exclusively comprised of samples collected at the end of lingonberry vegetation (4 October) and was characterized by the highest contents of anthocyanins, also higher than average contents of flavonol aglycones, phenolic acids, and their precursors. Winter persistent lingonberries collected on 28 December formed the fourth cluster and were distinguished by the highest contents of triterpenoids and phenolic acids precursors, but the lowest levels of flavan-3-ols, proanthocyanidins, arbutin derivatives, and phenolic acids.

### 3.2. Phytogeographical Patterns of Secondary Metabolites Accumulation in Lingonberry Fruits

**Figure 4:**
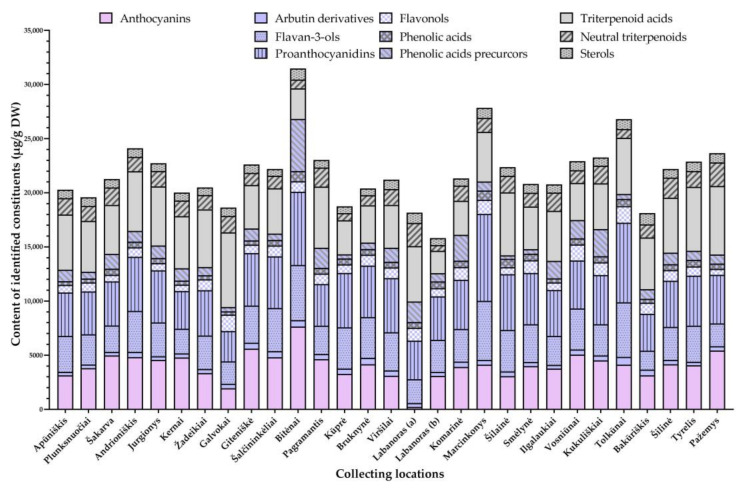
Contents of phenolics, and triterpenoids in lingonberries (μg/g DW), collected at different locations.

Contents of total identified constituents varied considerably among lingonberries from different collecting locations—between 15,819.5 μg/g DW in Labanoras (b) sample and 31,493.8 μg/g DW in Bitėnai sample. Significant differences in the chemical composition and accumulation of phenolics and triterpenoids (CV = 22.3%, and 20.8%, respectively) were determined and are presented in Figure 4.

#### 3.2.1. Phytogeographical Analysis of Phenolics

**Figure 5:**
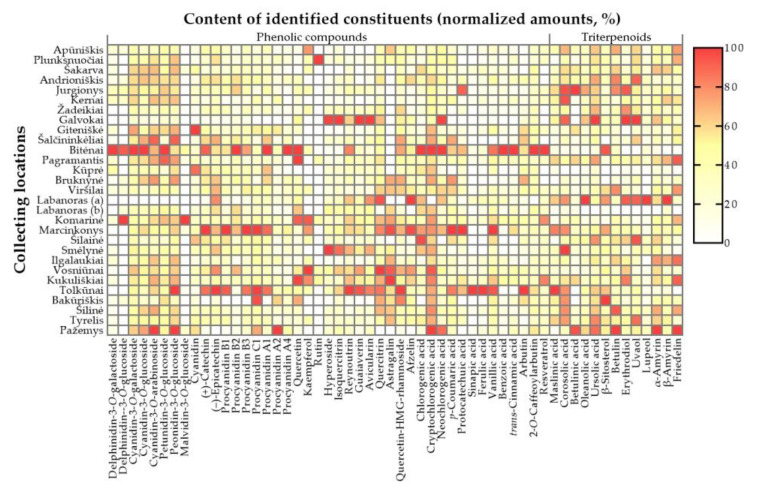
Heatmap estimating contents of individual constituents (%) in lingonberries based on their collecting locations.

Phenolic compounds constituted 50.7–85.2% of total identified constituents with the highest contribution and total contents determined in Bitėnai and Marcinkonys samples (26,821.65 and 21,059.69 μg/g DW, respectively). Accumulation patterns of individual phenolics are depicted in Figure 5 and Supplementary Tables S1–S5.

Anthocyanins contributed up to 24.7% of the total identified constituents in different samples. The greatest (*p* < 0.05) levels of most anthocyanins were determined in Bitėnai sample (7654.1 μg/g DW), followed by Giteniškė, and Pažemys samples. Besides habitat-dependent differences, the profile of anthocyanins was also genotype-dependent; 13.1-fold lower content *V. vitis-idaea* var. lingonberries of Labanoras (a) sample was found, compared to typical variety from the same collecting site (Labanoras (b)). Lingonberries from Galvokai were also distinguished by a considerably low content of total anthocyanins (1959.4 μg/g DW) and low contribution (0.8%) of minor anthocyanins. Levels of cyanidin glycosides did not correlate (*p* > 0.05) with contents of cyanidin aglycone, the greatest amounts of which were found in samples from Giteniškė and Kūprė forests. Negative correlations were determined between contents of particular anthocyanins in lingonberries to altitudes of their collecting locations (*r* ranged between −0.564 and −0.623, *p* < 0.05) and positive between contents of anthocyanins to macronutrients levels in the soil. The strongest correlations were found between contents of cyanidin glycosides to N, P, and K levels (*r* = 0.502–0.713, *p* < 0.05).

All lingonberries were characterized by the greatest contribution of proanthocyanidins (35.3–50.6%) out of phenolics group, with prevailing procyanidins B1 and B3 in all samples, except the Pažemys sample, which was distinguished by the predominance of procyanidin A2. Total contents of proanthocyanidins varied between 2808.3 μg/g DW and 8050.6 μg/g DW in samples of Galvokai and Marcinkonys, respectively. Levels of particular proanthocyanidins in wild lingonberries correlated negatively with altitudes of their collecting locations (*r* > −0.700, *p* < 0.05). Negative correlations between sum level of flavan-3-ols and altitudes of collecting locations were also determined (*r* = −0.650, *p* < 0.05). Out of flavan-3-ols, (+)-catechin accounted for 8.8–19.8% of total identified constituents and up to 26.1% of total phenolics. This compound prevailed in Marcinkonys and Bitėnai samples (5305.0 μg/g DW and 4974.6 μg/g DW, respectively), while contents of (−)-epicatechin were approx. 28.2-fold lower and predominated in the Tolkūnai sample (214.5 μg/g DW). Particular flavan-3-ols and proanthocyanidins in lingonberries negatively correlated with chloride levels in soil (*r* ranged between −0.603 and −0.671, *p* < 0.05).

The greatest total amount of identified flavonols was determined in the Tolkūnai sample (1549.8 μg/g DW) and the lowest content—in lingonberries from Pažemys forest (554.1 μg/g DW). The contribution of flavonols to total identified constituents and phenolics were in the ranges of 2.3–8.1% and 3.6–16.0%, respectively, with the highest contributions in the Galvokai sample. Quercitrin, followed by quercetin-HMG-rhamnoside and hyperoside, was the phytochemical marker present in most samples among flavonols. Only lingonberries of typical variety from Labanoras (b) and Pažemys were distinguished by superiority of avicularin, while *V. vitis-idaea* var. *leucocarpum* lingonberries (Labanoras (a)) were characterized by trace levels of quercetin-HMG-rhamnoside, but one of the highest amounts of quercitrin. Samples of Galvokai and Tolkūnai differ from others by the greatest contents of minor flavonols, whereas lingonberries from Kukuliškiai—by the highest total amount of flavonol aglycones. Amounts of them correlated negatively with longitudes and altitudes of collecting locations (*r* = −0.643 and −0.669, respectively, *r* < 0.05).

The complexity of phenolic acids prevailed in the Bitėnai sample (947.2 μg/g DW) and about 2.8-fold lower levels were detected in lingonberries from Galvokai, Bakūriškis, and Apūniškis forests. The highest contributions to total secondary metabolites and phenolics (3.5 and 5.5%, accordingly) were determined in the Šilainė sample. Chlorogenic acid was the main phenolic acid in most samples, except in Ilgalaukiai, Bakūriškis, and Pažemys samples, where the content of cryptochlorogenic acid was over chlorogenic acid. Contents of cryptochlorogenic were in a range of 67.6–188.2 μg/g DW, but were non-detectable in the unique Labanoras (a) sample. The quantitative profiles of phenolic acids precursors, especially benzoic acid, were highly dependent on habitat (CV = 75.6%) and varied between 326.5 μg/g DW and 4807.2 μg/g DW in lingonberries from Šilainė and Bitėnai, respectively, with a contribution up to 15.3% of total identified compounds and up to 19.0% of total phenolics. Contents of particular phenolic acids correlated negatively with latitudes of berries collecting locations (*r* ranged between −0.603 and −0.748, *p* < 0.05).

The greatest (*p* < 0.05) amount of simple phenolic—arbutin, which made up 1.3–2.8% of total identified compounds and 1.9–4.6% of total phenolics, was found in a sample of Tolkūnai forest (681.4 μg/g DW), and 2.4-fold lower content in Šakarva sample, while the highest contributions were found in Bakūriškis sample. 2-*O*-caffeoylarbutin prevailed in a sample from Bitėnai (70.3 μg/g DW) and only a traceable level of this compound was detected in *V. vitis-idaea* var. *leucocarpum* lingonberries. Contents of arbutin correlated negatively with a latitudinal gradient (*r* = −0.572, *p* < 0.05) and positively with P level in soil (*r* = 0.594, *p* < 0.05). One more phenolic—resveratrol was a minor lingonberry constituent and accumulated in quantifiable levels only in samples from Bitėnai, Komarinė, and Kukuliškiai (1.3–1.7 μg/g DW).

#### 3.2.2. Phytogeographical Analysis of Triterpenoids

Triterpenic compounds accounted for 14.8–49.3% of total lingonberry constituents with the lowest contribution in lingonberries from Bitėnai and the highest in Labanoras (a), Galvokai, and Pažemys samples, in which contents of triterpenoids were similar to that of phenolics (Figure 5 and Supplementary Tables S6 and S7). Overall, a negative significant correlation was found between levels of phenolics and triterpenoids (*r* = −0.523, *p* < 0.05). Content of triterpenoids was highly genotype-dependent because a 2.5-fold greater level of total identified triterpenoids was found in *V. vitis-idaea* var. *leucocarpum*, compared to typical variety.

Triterpenoid acids were found as a major subgroup of triterpenoids in all samples with ursolic acid being predominant, contributing up to 31.0% of total identified compounds and 44.5–62.8% of total triterpenoids. The highest (*p* < 0.05) content of ursolic acid was found in a sample of Galvokai forest (5777.5 μg/g DW), while 3.5-fold lower in a typical variety of Labanoras (b). Minor triterpenoid acids highly accumulated in a sample of *V. vitis-idaea* var. *leucocarpum* (17.9% to total identified triterpenoids)**,** followed by samples from Jurgionys and Galvokai. No significant correlations were found between geographical characteristics and contents of triterpenoid acids, but they correlated positively with macronutrients in soil. The strongest correlations were found between contents of corosolic and ursolic acids in lingonberries to N and Mg contents in soil (*r* = 0.587–0.669, *p* < 0.05).

The sum amount of neutral triterpenoids varied from 527.2 μg/g DW in a sample of Labanoras (b) to 2184.2 μg/g DW in Pažemys, and their profile was predominated by α-amyrin (195.1–851.2 μg/g DW); only *V. vitis-idaea* var. *leucocarpum* lingonberries were distinguished by notable superiority of lupeol (859.1 μg/g DW). Furthermore, this sample was characterized by the highest contribution of neutral triterpenoids to total identified compounds and to total triterpenoids (11.7 and 26.0%, respectively). Whereas, the lowest contribution to total compounds was found in the Bitėnai sample (2.6%) and total triterpenoids in lingonberries from Tolkūnai (11.8%). Moderate positive significant correlations were found between total content of neutral triterpenoids and N levels in soil (*r* = 0.576, *p* < 0.05) and exceptionally between content of amyrins and friedelin to latitudes of collecting locations (*r* = 0.564–0.636, *p* < 0.05).

The main sterol of lingonberries—β-sitosterol accounted for 2.8–5.8% of total identified constituents and up to 22.0% of total triterpenoids. The highest (*p* < 0.05) contributions and amounts of this compound were determined in samples from Bakūriškis and Bitėnai (1055.1 μg/g DW and 1028.2 μg/g DW, respectively). Contents of sterols in lingonberries from Bitėnai and Tolkūnai even surpassed levels of neutral triterpenoids. Whereas, samples from Kūprė, Šalčininkėliai, and Bruknynė stood out by the lowest (*p* < 0.05) level of β-sitosterol (613.8–620.1 μg/g DW). Levels of β-sitosterol correlated weakly but significantly with Ca contents in soil (*r* = 0.478, *p* < 0.05).

#### 3.2.3. Principal Component Analysis of Phytogeographical Data

Principal component analysis (PCA) was carried out in order to determine the relative distribution of lingonberry fruits obtained from different geographical regions on the basis of phenolics and triterpenoids composition. Four main derived principal components explained 70.9% of the total variance. The first principal component (PC1) had the highest portion of variance (29.9%) and was associated with positive loadings of identified proanthocyanidins, flavan-3-ols, phenolic acids, arbutin, and quercetin-HMG-rhamnoside out of flavonols group, and negative loadings of neutral triterpenoids. The second component (PC2), explaining 16.7% of the variance, positively correlated with contents of triterpenoid acids, neutral triterpenoids, and sterols. The third principal component (PC3) accounted for 13.8% of the total variance and was mainly negatively correlated with identified anthocyanins and positively with flavonol glycosides. The fourth component (PC4) represented 10.5% of the total variance and benzoic and *trans*-cinnamic acids. Furthermore, flavonol aglycones were the main variables contributing to its definition.

**Figure 6:**
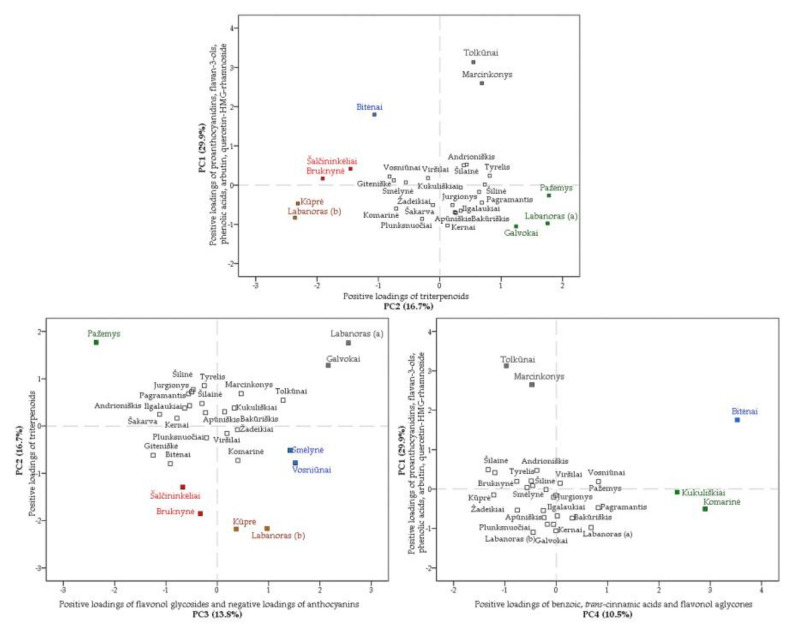
PCA scores estimating contents of phenolics and triterpenoids in lingonberries based on their collecting locations.

The PCA score plots of fruit samples showed their arrangement into distinct groups and most distanced from others were highlighted in Figure 6. Lingonberries collected from Tolkūnai and Marcinkonys were grouped at the positive side of first and second principal components and distinguished by the highest contents of flavan-3-ols, procyanidins A1, B1, B3, C1; *p*-coumaric; vanillic; and protocatechuic acid, as well as high loadings of triterpenoids (maslinic, corosolic acids, and β-sitosterol). These samples were collected from the same region of South Lithuania and were characterized by higher than average altitudes and one of the lowest latitudes. Samples collected from the North or North-East Lithuania, namely Galvokai, Pažemys, and Labanoras (a), were located at the positive side of PC2 due to high levels of triterpenoid acids and neutral triterpenoids. However, lingonberries from Pažemys were characterized as the anthocyanins-rich sample with the highest contents of cyanidin-3-*O*-arabinoside and peonidin-3-*O*-glucoside, while anthocyanins in *V. vitis-idaea var. leucocarpum* (Labanoras (a)) and Galvokai samples were considered as minor contributors, so these samples were located at different sides of PC3. Despite genotype-dependent differences, levels of N and K in the Pažemys soil sample notably surpassed levels in Labanoras (a) and Galvokai samples. Šalčininkėliai and Bruknynė samples from Southeast Lithuania were characterized by the lowest contents of sterols, but higher than average values of cyanidin glycosides and were grouped at the negative sides of second and third principal components. Additionally, these samples were distinguished by lower-than-average pH values of soil and the predominance of *Picea abies* (L.) H. Karst trees in collecting sites. A typical variety sample of Labanoras (b) and sample from the Kūprė forest in West Lithuania differ from others by one of the lowest contents of all macronutrients in soil and were grouped at the negative side of PC2 and positive of PC3 mainly because of low loadings of all identified triterpenoids and lower than average levels of anthocyanins. Lingonberries from Smėlynė and Vosniūnai (both from North-East Lithuania) were predominated by identified flavonol glycosides and were grouped at the positive side of PC3. Samples of Kukuliškiai and Komarinė collected from West Lithuania were located along the positive side of PC4 and were distinguished by low altitudes and longitudes and higher than the average values of total macronutrients in soil. The highest amounts of quercetin, kaempferol, and high loadings of phenolic acids precursors were found in these lingonberries. The Bitėnai sample from West Lithuania was distanced from all others at the positive side of the fourth and first principal components, but was negative of second and was characterized by the phytochemical marker of benzoic acid, the highest contents of most phenolics, as well as the lowest of identified triterpenoid acids and neutral triterpenoids. The collecting location of Bitėnai differed from others by the lowest altitude.

## 4. Discussion

The phytochemical profile of lingonberry fruits consists of a multi-component mixture of different origin compounds, which exhibit a broad spectrum of biological activities, therefore are of great interest in the pharmaceutical industry [14,30]. Our study provided further evidence for high contents and a wide diversity of bioactive lingonberry components. As far as we are aware, this is the first comprehensive report concerning the qualitative and quantitative simultaneous analysis of 34 phenolics and 13 triterpenoids in lingonberry fruits. Cyanidin-3-*O*-galactoside, which possess strong antioxidant activity [31]; (+)-catechin, procyanidins B1, B3, A1, which have been associated with urinary tract health [32]; benzoic acid, known for antimicrobial activity [33]; and ursolic acid with clinical interest due to its anticancer and neuromodulatory potential [34] were determined as major phytochemical markers of lingonberries (mean values exceeded 1000.0 μg/g DW). This is in good agreement with previous studies [35,36,37], wherein these predominant compounds were highlighted. A significant variation of identified components was found in the present study, suggesting a strong impact of phenological, geographical, and other related factors. Plants constantly respond to these factors in specific conditions, resulting in biosynthesis and chemical composition of secondary metabolites changes [6].

Phenological variation in secondary metabolites has been documented repeatedly in numerous plant species and is considered to be linked to the metabolic and physiological changes during the plant life cycle [4,5,23,38]. According to our results, a remarkable increase in contents of anthocyanins and phenolic acids precursors was observed at the end of lingonberry fruits vegetation, but the highest level of other identified compounds—proanthocyanidins, flavan-3-ols, flavonols, arbutin derivatives, phenolic acids, and high contents of triterpenoids—was found in unripe fruits samples. A similar conclusion was reached by Karppinen et al. [22], who reported that proanthocyanidins, flavonols, and phenolic acids are prevailing components of *Vaccinium* berries at the early stages of their development. These results may serve for phytopharmaceuticals development and bioactive compounds isolation purposes when the better yield of particular components is needed. However, the observed variance should be understood not strictly based on accumulation and degradation of identified phenolics and triterpenoids, but as a result of biosynthesis of all primary and secondary metabolites and their different contribution during phenophases. Since lingonberries’ sugar accumulation peaks at the berry maturity stage [39], the lower contribution and levels of other constituents are expected.

Significant negative correlations determined between amounts of proanthocyanidins and anthocyanins also between phenolic acids and their precursors during lingonberries vegetation could be explained by the closely related upstream biosynthesis pathways and allocation of these compounds [40]. Our findings corroborate with the report of Teixeira et al. [41], who announced that the accumulation of anthocyanins in berries tends to reach its maximum just in the latest phases of fruit maturation when the synthesis of proanthocyanidins is already stopped and study of Gruz et al. [42], wherein the concentrations of phenolic acids decreased as the ripening of tested fruits progressed. Even though the lingonberry plant can withstand cold temperatures, fruits are not persistent and typically their vegetation ends in the first half of autumn [10]. However, due to the warm 2019 winter in Lithuania, we succeeded in finding a small sample of lingonberries on 28 December. This work demonstrated intensified continuous biosynthesis of benzoic acid and most triterpenoids and indicated acclimation to harsh weather conditions, while suspended metabolic activity and decay processes of other identified compounds, especially flavan-3-ols, proanthocyanidins, and arbutin derivatives. An initial high content of triterpenoids at early stages of lingonberries formation, which sharply decreases and re-increases at the end of the vegetation, is explained by triterpenoids redirection during fruit maturation to other locations in fruits, like seeds or into other organs [36].

Plants’ phenological stages depend on surrounding climatic conditions and are regulated by the interactions with environmental cues [5,23]. The formation of lingonberry fruits in Lithuania is expected in August [26], but actually, formation starts earlier as presented in our study, hence reported phenological phases are likely to be slightly shifted already due to the changes in climate over time and adaptation processes. Environmental factors, namely sunlight exposure, temperature, and water intake, can significantly modify the content and composition of bioactive components by affecting the expression of structural and regulatory genes [24]. Higher temperature and longer sunshine duration profoundly increased contents of flavan-3-ols and proanthocyanidins in tested lingonberries. Since latitudinal, longitudinal, and altitudinal gradients negatively correlate to temperature and sunshine duration [43], our results from the study of wild lingonberries growing at different locations also revealed the positive impact of these environmental cues on the accumulation of anthocyanins, proanthocyanidins, flavan-3-ols, phenolic acids, arbutin, and flavonol aglycones. Mikulic-Petkovsek et al. [44] announced similar correlations between environmental factors and bilberry’s inner quality parameters and reported that bilberries growing in locations with higher light intensity accumulate higher levels of total sugars, anthocyanins, flavan-3-ols, and hydroxycinnamic acids. A study by Dincheva and Badjakov [45] showed that higher contents of total phenolics and anthocyanins were determined in samples harvested from habitats exposed to the sun in comparison with lingonberries grown in the shadow. Several other researchers confirmed the positive effect of light and temperature on anthocyanins and other phenolics contents in fruits [46,47]. Contrary to these findings, cold acclimation and harsh weather conditions had a positive impact on the content of phenolics in lingonberry leaves [48]. This appears to be due to different adaptability and defense mechanisms to climatic conditions, as well as metabolites’ allocation via organs after the reproductive growth period [49]. Concerning environmental and geographical factor’s impact on the contents of triterpenoids, the accumulation of neutral triterpenoids increased progressively with increasing latitudes of lingonberries collecting locations. The cold acclimation phenomenon of neutral triterpenoids was confirmed by negative correlations to air temperature and sunshine duration. Since triterpenoids are known for antifungal properties [50], biosynthesis of them during cold and rainy seasons could be intensified in the response to pathogens infections and pressure of other biotic agents.

Soil health is important for the luxuriant growth of the plants, hence nutritional deficiency or excess can affect the accumulation of bioactive compounds profoundly and lead to multiple deficiencies [25]. In situations when macronutrients are not present in sufficient amounts, plants tend to develop adaptive and flexible strategies for the acquisition, like macronutrients’ distribution throughout the plant [51]. Our results demonstrated a positive impact of particular macronutrients in soil for the biosynthesis of bioactive compounds in lingonberry fruits, especially for the accumulation of anthocyanins and triterpenoids. Nitrogen, phosphorus, and potassium are regarded as primary plant nutrients, essential for plant development [52], and according to obtained strongest correlations can be considered as the main required macronutrients for the biosynthesis of most identified lingonberry compounds. Negative correlations found in our study to chloride levels in soil suggest unfavorable salinity, which is regarded as one of the most brutal abiotic stresses [53]. For proper growth and development, *Vaccinium* species demand relatively low soil pH as an important chemical parameter influencing nutrients availability [54]. In line with a previous study [55], our present findings suggest that relatively low soil pH values may be preferable for the biosynthesis of anthocyanins in lingonberries.

Metabolites profiling among samples from different locations allows detection of chemical diversity and provides functional information on the metabolic phenotypes of plants [56]. Present findings on wild lingonberries from different regions of Lithuania showed arrangement into diverse populations, distinguished by predominant components and disparity in the contents of individual compounds. Adaptive phenotypic plasticity and possible gene flow in plants are the reasons for chemical diversity among the same plant species growing in different conditions [57,58]. Thus, our determined chemophenetic differences could be explained by the variation of climatic and geographical conditions discussed above, their impact related to the habitats of lingonberries, and genetic differentiation. Previously, few studies showed that *Vaccinium* berries originating from different geographic regions and environments differ from each other in terms of bioactive compound quantity and biological activity, consequently indicating that geographical origin must be considered in case of selection of new perspective cultivars and domestication purposes [59,60,61,62].

Accumulation of bioactive lingonberry components appears to be regulated by the interactions to surrounding geographical conditions and defense mechanisms to physiological and environmental changes, which are overall related to genetic origin [63,64]. Our study examined one variety of lingonberries—*V. vitis-idaea* var. *leucocarpum*, which is included in the National Genetic Resources of Lithuania. In 1993, lingonberries of this variety were found in the Labanoras, forest of Švenčionėliai district, and so far no more floras have been announced as natural habitats of this variety [26]. *V. vitis-idaea* var. *leucocarpum* was previously emphasized due to very low contents of anthocyanins [27], high fructose and glucose content in fruits [39], and strong reducing and chelating activities of leaves extracts [65]. Current results have provided novel insights about the unique phenolic profile of this variety berries and considerable richness of neutral triterpenoids and triterpenoid acids, thus suggesting strong triterpenoids-related health benefits.

## 5. Conclusions

The qualitative and qualitative composition of phenolics and triterpenoids in lingonberry fruits varied significantly during growth stages and were strongly dependent on genetic and geographical origin, environmental, and edaphic conditions. The highest concentrations of anthocyanins and phenolic acids precursors were found in fully ripe lingonberries, while proanthocyanidins, flavan-3-ols, flavonols, phenolic acids, and arbutin derivatives prevailed in the early stages of berry vegetation. Triterpenoids accumulated highly in unripe lingonberries as well but also showed intensified continuous biosynthesis after vegetation season in winter. Chemophenetic differences among lingonberries were found in the present study, most likely due to variations in the climatic and geographical conditions of their collecting localities. Lower latitudinal, longitudinal, and altitudinal gradients with sufficient main macronutrients levels in soil, higher solar radiation, and temperature seem to be favorable growing conditions for the accumulation of most lingonberry constituents, except biosynthesis of triterpenoids and phenolic acids precursors with tendencies towards harsh weather acclimation. Consideration of these factors is recommended to obtain higher yields of lingonberry fruits with considerable greater amounts of particular bioactive components, eligible in the production of functional food and pharmaceuticals.

## Figures and Tables

**Table 1:** Geographical coordinates and edaphic characteristics of the lingonberry collecting locations in Lithuania.

No.	Forest	Geographical Parameters		Soil Properties							
		Altitude, m	Coordinates	pH	EC, mS/cm	N, mg/L	P, mg/L	K, mg/L	Ca, mg/L	Mg, mg/L	Cl, mg/L
1	Žadeikiai	52	56°00′52.8″ N 24°28′03.2″ E	3.0	0.17	6	2	36	12	5	18
2	Vosniūnai	77	55°53′59.4″ N 24°41′08.8″ E	3.1	0.67	27	7	65	41	14	18
3	Galvokai	80	56°03′28.4″ N 24°53′30.5″ E	3.1	0.38	40	3	29	19	7	23
4	Viršilai	102	56°05′37.2″ N 25°18′48.8″ E	3.1	0.64	28	7	50	55	8	18
5	Plunksnuočiai	107	56°04′32.8″ N 25°29′41.1″ E	3.4	0.36	16	2	32	15	5	18
6	Ilgalaukiai	107	55°59′20.6″ N 25°25′48.2″ E	2.9	0.47	30	8	51	57	8	19
7	Apūniškis	110	56°00′40.6″ N 25°31′29.4″ E	4.2	1.10	116	6	48	60	23	18
8	Bakūriškis	123	56°03′46.9″ N 25°40′44.1″ E	2.9	0.83	39	10	66	61	15	23
9	Andrioniškis	111	55°36′03.3″ N 25°02′20.4″ E	3.6	0.56	50	10	91	24	6	18
10	Pažemys	171	55°39′45.0″ N 25°59′43.1″ E	3.5	0.94	114	3	87	23	9	23
11	Giteniškė	173	55°35′45.1″ N 26°07′53.7″ E	4.1	0.19	7	0.2	20	9	4	18
12	Smėlynė	187	55°23′56.0″ N 26°08′55.3″ E	3.9	0.14	60	6	64	26	5	15
13	Šakarva	149	55°18′41.6″ N 26°03′47.4″ E	3.6	1.68	6	2	31	10	4	23
14	Labanoras	165	55°09′25.1″ N 25°48′24.9″ E	3.3	0.27	12	0.9	20	12	4	18
15	Šalčininkėliai	171	54°22′40.3″ N 25°22′30.6″ E	3.1	0.90	73	17	101	24	9	20
16	Bruknynė	189	54°20′55.1″ N 25°23′12.0″ E	3.2	0.95	80	17	113	26	11	20
17	Jurgionys	139	54°27′46.2″ N 24°29′58.3″ E	3.3	1.11	89	28	116	25	18	22
18	Tolkūnai	145	54°16′26.2″ N 24°24′25.3″ E	3.8	0.52	70	18	88	31	12	19
19	Marcinkonys	137	54°04′09.9″ N 24°26′06.1″ E	3.2	0.21	9	1	26	12	4	12
20	Šilainė	134	54°04′48.8″ N 23°42′40.9″ E	2.9	0.29	11	2	30	17	6	23
21	Bitėnai	22	55°03′51.1″ N 22°02′50.7″ E	3.2	0.99	27	4	36	13	6	17
22	Šilinė	39	55°11′10.5″ N 22°18′52.0″ E	3.3	0.25	57	12	45	13	6	18
23	Komarinė	47	55°11′12.2″ N 22°27′04.3″ E	2.9	0.62	48	6	56	22	8	18
24	Pagramantis	89	55°23′28.6″ N 22°13′04.4″ E	3.2	0.85	105	12	76	32	15	20
25	Tyrelis	59	55°19′22.0″ N 22°10′04.1″ E	3.2	0.97	166	10	96	49	16	20
26	Kūprė	145	55°34′30.2″ N 22°48′57.3″ E	3.8	0.22	8	0.4	15	9	4	23
27	Kukuliškiai	34	55°46′55.7″ N 21°05′22.3″ E	4.8	1.70	52	7	61	10	13	20
28	Kernai	44	56°13′45.8″ N 21°29′18.5″ E	4.0	1.13	185	13	113	76	27	23

**Table 2:** Contents of phenolics (μg/g DW ± SD) in lingonberries during different phenological stages. Values marked with * in the same row indicate the highest (*p* < 0.05) amounts in samples. ND—not detected, NQ—not quantified.

Phenolic Compound	1 July	15 July	29 July	11 August	24 August	6 September	20 September	4 October	28 December
Delphinidin-3-*O*-galactoside	ND	ND	ND	NQ	8.2 ± 0.1	10.4 ± 0.3	14.9 ± 0.2	34.1 ± 1.1 *	9.6 ± 0.2
Delphinidin-3-*O*-glucoside	ND	ND	ND	ND	NQ	NQ	NQ	NQ	NQ
Cyanidin-3-*O*-galactoside	5.9 ± 0.1	10.3 ± 0.3	119.6 ± 0.5	1411 ± 35.2	1464.6 ± 24.0	2147.1 ± 6.5	2578.6 ± 84.2	3135.7 ± 61.0 *	2105.1 ± 87.9
Cyanidin-3-*O*-glucoside	ND	ND	6.4 ± 0.0	70.9 ± 3.0	115.1 ± 4.3	191.8 ± 0.5	195.0 ± 2.9	229.2 ± 10.3 *	191.6 ± 3.5
Cyanidin-3-*O*-arabinoside	ND	ND	1.7 ± 0.0	81.2 ± 3.0	127.1 ± 3.9	274.7 ± 0.6	299.1 ± 13.2	400.2 ± 10.6 *	325.6 ± 9.5
Petunidin-3-*O*-glucoside	ND	ND	ND	ND	10.5 ± 0.1	11.0 ± 0.1	11.3 ± 0.4	16.1 ± 0.8 *	10.9 ± 0.1
Peonidin-3-*O*-glucoside	ND	ND	NQ	10.4 ± 0.4	11.6 ± 0.2	23.0 ± 0.3	25.3 ± 1.4	31.2 ± 0.3 *	28.7 ± 1.0
Malvidin-3-*O*-glucoside	ND	ND	ND	ND	ND	ND	ND	NQ	NQ
Cyanidin	ND	ND	ND	7.5 ± 0.3	8.2 ± 0.3	10.3 ± 0.5	11.4 ± 0.2	15.4 ± 0.5 *	8.6 ± 0.3
Sum of anthocyanins	5.9	10.3	127.7	1581.1	1745.4	2668.3	3135.6	3862.0	2680.2
(+)-Catechin	7697.8 ± 17.7 *	7121.9 ± 38.5	4217.2 ± 6.6	4662.6 ± 94.6	3744.3 ± 22.3	3635.7 ± 101.3	3482.8 ± 37.3	4076.6 ± 146.0	874.9 ± 4.7
(−)-Epicatechin	441.6 ± 0.2 *	293.6 ± 7.9	182.1 ± 11.0	181.1 ± 3.9	151.9 ± 5.9	129.7 ± 5.3	118.1 ± 2.7	172.1 ± 3.6	51.2 ± 1.0
Sum of flavan-3-ols	8139.4	7415.5	4399.3	4843.7	3896.2	3765.4	3600.9	4248.7	926.1
Procyanidin A1	2149.1 ± 10.3 *	1590.3 ± 10.1	965.6 ± 31.9	956.9 ± 2.2	908.5 ± 10.9	817.4 ± 48.6	801.6 ± 22.2	998.6 ± 15.4	361.8 ± 17.1
Procyanidin A2	705.3 ± 18 *	449.0 ± 0.3	286.4 ± 9.5	275.6 ± 9.4	275.5 ± 11.5	272.2 ± 8.9	220.8 ± 5.7	381.5 ± 17.9	349.2 ± 3.7
Procyanidin A4	158.0 ± 3.4 *	74.6 ± 7.2	17.2 ± 0.3	15.1 ± 0.5	14.8 ± 0.2	14.4 ± 0.2	12.3 ± 0.5	14.0 ± 0.1	2.8 ± 0.2
Procyanidin B1	2442.7 ± 11.8 *	2230.5 ± 6.5	1920.1 ± 80.0	1670.9 ± 8.0	1425.7 ± 29.6	1418.9 ± 13.0	1284.7 ± 19.5	1313.3 ± 9.9	572.4 ± 19.2
Procyanidin B2	1117.7 ± 25.1 *	695.0 ± 32.3	598.6 ± 21.4	455.9 ± 20.6	470.8 ± 2.8	460.0 ± 18.6	351.7 ± 14.9	519.3 ± 7.5	154.7 ± 4.4
Procyanidin B3	5389.0 ± 14.9 *	3703.5 ± 57.9	3167.4 ± 9.6	1805.8 ± 43.2	1376.9 ± 63.7	1252.1 ± 26.3	1159.8 ± 41.6	1162.0 ± 43.4	207.3 ± 7.9
Procyanidin C1	1515.3 ± 25.1 *	1065.8 ± 8.3	633.6 ± 21.4	576.3 ± 16.6	493.9 ± 12.7	467.8 ± 4.8	425.2 ± 4.3	489.9 ± 12.2	367.0 ± 6.7
Sum of proanthocyanidins	13477.1	9808.7	7588.9	5756.5	4966.1	4702.8	4256.1	4878.6	2015.2
Quercetin	39.4 ± 0.2 *	40.5 ± 0.4 *	38.5 ± 0.8	37.5 ± 1.9	35.9 ± 1.4	34.3 ± 0.0	33.5 ± 0.1	43.3 ± 1.7 *	43.0 ± 0.1 *
Kaempferol	1.8 ± 0.0 *	1.8 ± 0.0 *	NQ	NQ	NQ	NQ	NQ	1.9 ± 0.1 *	1.7 ± 0.1 *
Rutin	85.6 ± 0.8 *	81.2 ± 0.3	3.0 ± 0.0	ND	ND	ND	ND	ND	ND
Hyperoside	196.7 ± 1.3 *	177.4 ± 0.1	120.2 ± 4.5	91.2 ± 0.5	79.1 ± 1.7	78.3 ± 2.9	72.5 ± 0.6	66.8 ± 2.1	65.7 ± 1.0
Isoquercitrin	716.2 ± 3.0	1153.7 ± 30.5 *	566.4 ± 18.0	149.3 ± 5.4	85.7 ± 2.5	56.8 ± 2.0	51.6 ± 1.6	43.9 ± 0.9	39.2 ± 0.6
Reynoutrin	57.0 ± 0.1 *	49.9 ± 0.2	35.4 ± 1.0	29.1 ± 0.1	28.9 ± 0.9	32.9 ± 0.6	33.4 ± 0.5	39.9 ± 0.6	46.5 ± 1.0
Guaiaverin	42.6 ± 1.5 *	45.0 ± 0.9 *	27.8 ± 1.2	19.9 ± 0.1	17.1 ± 0.2	17.0 ± 0.4	16.9 ± 0.3	14.7 ± 0.8	21.0 ± 0.6
Avicularin	214.5 ± 0.2 *	199.0 ± 0.1	114.8 ± 5.6	105.5 ± 0.8	100.4 ± 2.3	94.4 ± 0.9	92.6 ± 8.3	90.8 ± 3.9	127.5 ± 2.4
Quercitrin	627.0 ± 3.2 *	514.0 ± 4.2	456.1 ± 14.7	332.3 ± 2.7	329.9 ± 14.4	303.5 ± 15.2	281.0 ± 7.2	272.8 ± 9.3	375.8 ± 7.0
Quercetin-HMG-rhamnoside	384.1 ± 2.7 *	346.0 ± 1.6	253.8 ± 0.8	206.3 ± 0.1	186.3 ± 3.6	172.2 ± 10.1	149.8 ± 2.9	145.7 ± 2.1	227.0 ± 4.2
Astragalin	5.6 ± 0.2 *	5.5 ± 0.1 *	5.4 ± 0.1 *	5.2 ± 0.1	5.0 ± 0.1	5.0 ± 0.2	4.6 ± 0.2	4.7 ± 0.1	5.4 ± 0.3 *
Afzelin	12.2 ± 1.2 *	12.0 ± 1.3 *	11.4 ± 0.5 *	10.7 ± 0.1	8.3 ± 0.4	7.9 ± 0.2	6.9 ± 0.1	7.1 ± 0.2	9.3 ± 0.4
Sum of flavonols	2382.7	2626.0	1632.8	987.0	876.6	802.3	742.8	731.6	962.1
Benzoic acid	92.9 ± 1.7	110.7 ± 4.3	191.8 ± 7.1	338.8 ± 7.9	573.3 ± 26.6	1126.0 ± 13.7	1536.8 ± 65.1	2633.1 ± 62.0	3713.6 ± 52.4 *
*trans*-Cinnamic acid	8.1 ± 0.0	10.5 ± 0.9	11.6 ± 0.1	11.9 ± 0.1	13.9 ± 0.4	16.2 ± 0.7	17.5 ± 0.1	27.2 ± 1.7 *	24.8 ± 0.0 *
Sum of phenolic acids precursors	101.0	121.2	203.4	350.7	587.2	1142.2	1554.3	2660.3	3738.4
Vanillic acid	65.0 ± 0.7 *	39.3 ± 1.1	21.0 ± 0.0	14.5 ± 0.3	11.2 ± 0.6	15.7 ± 0.9	19.4 ± 0.9	22.3 ± 0.8	44.9 ± 1.2
Protocatechuic acid	104.1 ± 0.1 *	99.6 ± 0.5 *	96.5 ± 1.1	90.0 ± 1.9	89.5 ± 1.9	84.5 ± 3.4	78.1 ± 2.3	102.5 ± 2.8 *	96.8 ± 1.0
Chlorogenic acid	464.8 ± 2 *	311.0 ± 0.1	196.0 ± 8.2	178.4 ± 5.3	172.9 ± 7.6	166.1 ± 6.3	124.6 ± 6.2	168.3 ± 5.6	103.7 ± 2.2
Cryptochlorogenic acid	161.3 ± 5.4 *	119.0 ± 1.4	84.0 ± 0.6	83.2 ± 1.8	77.7 ± 0.2	75.0 ± 2.1	71.6 ± 3.0	103.3 ± 1.8	70.2 ± 0.4
Neochlorogenic acid	42.9 ± 2.4 *	38.1 ± 0.4 *	33.3 ± 0.9	31.3 ± 0.6	29.3 ± 0.2	16.8 ± 0.3	13.4 ± 0.2	19.5 ± 0.3	15.3 ± 0.4
*p*-Coumaric acid	203.7 ± 1.8 *	118.0 ± 2.8	108.0 ± 4.5	69.8 ± 0.7	61.3 ± 1.2	59.2 ± 1.4	39.8 ± 1.2	65.1 ± 3.0	39.4 ± 0.5
Sinapic acid	8.1 ± 0.2 *	3.8 ± 0.1	NQ	NQ	NQ	2.3 ± 0.1	2.5 ± 0.2	4.4 ± 0.4	6.2 ± 0.2
Ferulic acid	16.4 ± 0.3 *	12.5 ± 0.1	7.7 ± 0.2	7.7 ± 0.2	5.7 ± 0.1	9.5 ± 0.2	9.7 ± 0.3	12.1 ± 0.5	9.6 ± 0.3
Sum of phenolic acids	1066.3	741.3	546.5	474.9	447.6	429.1	359.1	497.5	386.1
Arbutin	1518.7 ± 10.3 *	1117.9 ± 2.6	603.6 ± 29.5	519.5 ± 9.9	518.2 ± 4.4	445.4 ± 5.0	342.1 ± 1.3	430.2 ± 6.7	69.3 ± 3.2
2-*O*-caffeoylarbutin	128.7 ± 1.1 *	83.4 ± 0.3	39.0 ± 1.1	38.0 ± 0.4	36.2 ± 1.2	28.8 ± 1.7	24.9 ± 0.3	31.8 ± 1.8	14.1 ± 0.1
Sum of arbutin derivatives	1647.4	1201.3	642.6	557.5	554.4	474.2	367.0	462.0	83.4
Resveratrol (Stilbene)	0.5 ± 0.0 *	ND	ND	ND	ND	ND	ND	ND	ND
Total identified phenolics	26,820.3	21,924.3	15,141.2	14,551.3	13,073.4	13,984.3	14,015.8	17,340.6	10,791.4

**Table 3:** Contents of triterpenoids (μg/g DW ± SD) in lingonberries during different phenological stages. Values marked with * in the same row indicate the highest (*p* < 0.05) amounts in samples. ND—not detected, NQ—not quantified.

Triterpenoid	1 July	15 July	29 July	11 August	24 August	6 September	20 September	4 October	28 December
Maslinic acid	23.6 ± 1.1	13.4 ± 0.0	12.0 ± 0.2	11.6 ± 0.4	9.9 ± 0.3	9.2 ± 0.1	17.2 ± 0.1	25.7 ± 0.7	35.2 ± 1.0 *
Corosolic acid	39.8 ± 1.0	31.3 ± 0.8	30.3 ± 0.5	26.9 ± 0.7	26.2 ± 1.5	25.0 ± 0.7	33.3 ± 0.7	33.6 ± 0.3	66.4 ± 6.3 *
Betulinic acid	NQ	NQ	NQ	NQ	NQ	NQ	NQ	3.12 ± 0.21	12.38 ± 0.56 *
Oleanolic acid	1031.7 ± 0.9 *	818.9 ± 2.0	791.8 ± 6.3	690.9 ± 98.7	659.8 ± 35.9	497.3 ± 4.9	537.5 ± 10.9	555.3 ± 5.3	1109.3 ± 21.8 *
Ursolic acid	5067.3 ± 32.9 *	4636.1 ± 14.6	4019.3 ± 53.6	3677.5 ± 13.6	3383.1 ± 65.0	2879.7 ± 35.9	3071.1 ± 21.6	3164.0 ± 22.5	4920.6 ± 34.5 *
Sum of triterpenoid acids	6162.4	5499.7	4853.4	4406.9	4079.0	3411.2	3659.1	3781.7	6143.9
Betulin	368.0 ± 12.9	298.3 ± 14.3	291.3 ± 5.3	141.3 ± 1.3	149.4 ± 1.2	236.9 ± 7.1	244.5 ± 5.5	466.6 ± 11.9	930.4 ± 28.3 *
Erythrodiol	30.5 ± 1.4 *	6.0 ± 0.1	6.4 ± 0.4	2.2 ± 0.1	6.7 ± 0.2	6.2 ± 0.2	8.8 ± 0.6	11.4 ± 0.2	27.7 ± 1.0 *
Uvaol	64.3 ± 1.6	44.9 ± 1.5	36.4 ± 3.2	29.6 ± 0.2	28.7 ± 0.4	30.4 ± 0.6	34.5 ± 0.3	35.9 ± 1.4	80.5 ± 0.3 *
Lupeol	242.4 ± 4.4 *	210.8 ± 1.2	174.5 ± 0.3	90.4 ± 2.2	108.9 ± 3.6	203.7 ± 0.4	204.0 ± 8.6	204.4 ± 4.8	218.1 ± 2.3
α-Amyrin	686.5 ± 9.1	593.3 ± 30.8	482.0 ± 2.8	435.2 ± 6.6	476.4 ± 9.3	504.9 ± 12.4	558.2 ± 13.0	599.5 ± 12.6	979.8 ± 125.1 *
β-Amyrin	166.6 ± 5.9 *	155.7 ± 0.9 *	152.1 ± 2.3	138.1 ± 1.7	114.8 ± 4.4	103.6 ± 4.4	111.5 ± 2.0	119.7 ± 1.6	150.5 ± 4.1
Friedelin	471.3 ± 13.0 *	444.8 ± 16.0	359.3 ± 11.3	180.1 ± 4.2	189.2 ± 4.0	215.6 ± 9.4	314.6 ± 3.6	353.0 ± 9.2	437.6 ± 15.1
Sum of neutral triterpenoids	2029.6	1753.8	1502.0	1016.9	1074.1	1301.3	1476.1	1790.5	2824.6
β-Sitosterol (Sterol)	1087.7 ± 11.5 *	999.8 ± 22.5	959.1 ± 31.3	821.7 ± 6.7	829.7 ± 0.7	830.5 ± 4.6	879.4 ± 0.9	940.7 ± 4.6	1105.7 ± 14.5 *
Total identified triterpenoids	9279.7	8253.2	7314.5	6245.6	5982.7	5543.0	6014.6	6512.9	10,074.2

## Data Availability

All data generated during this study are included in this article.

## Supplementary Materials

The following are available online at https://www.mdpi.com/article/10.3390/foods10102243/s1, Figure S1: Climatic data in Lithuania during lingonberry vegetation in 2019; Table S1: Contents of anthocyanins (μg/g DW ± SD) in lingonberries, collected at different locations; Table S2: Contents of flavan-3-ols and proanthocyanidins (μg/g DW ± SD) in lingonberries, collected at different locations; Table S3: Contents of flavonol glycosides (μg/g DW ± SD) in lingonberries, collected at different locations; Table S4: Contents of phenolic acids (μg/g DW ± SD) in lingonberries, collected at different locations; Table S5: Contents of simple phenolics, phenolic acids precursors, flavonol aglycones, and stilbenes (μg/g DW ± SD) in lingonberries, collected at different locations; Table S6: Contents of triterpenoid acids and sterols (μg/g DW ± SD) in lingonberries, collected at different locations; Table S7: Contents of neutral triterpenoids (μg/g DW ± SD) in lingonberries, collected at different locations.
