# Supplementary material for: Vaccinium vitis-idaea L. Fruits: Chromatographic Analysis of Seasonal and Geographical Variation in Bioactive Compounds
Source: Foods. 2021 Sep 22;10(10):2243. doi: 10.3390/foods10102243 (PMC8535033; doi:10.3390/foods10102243)
Supplement: Supplementary file 1 [file foods-10-02243-s001.zip › Supplementary material.pdf]

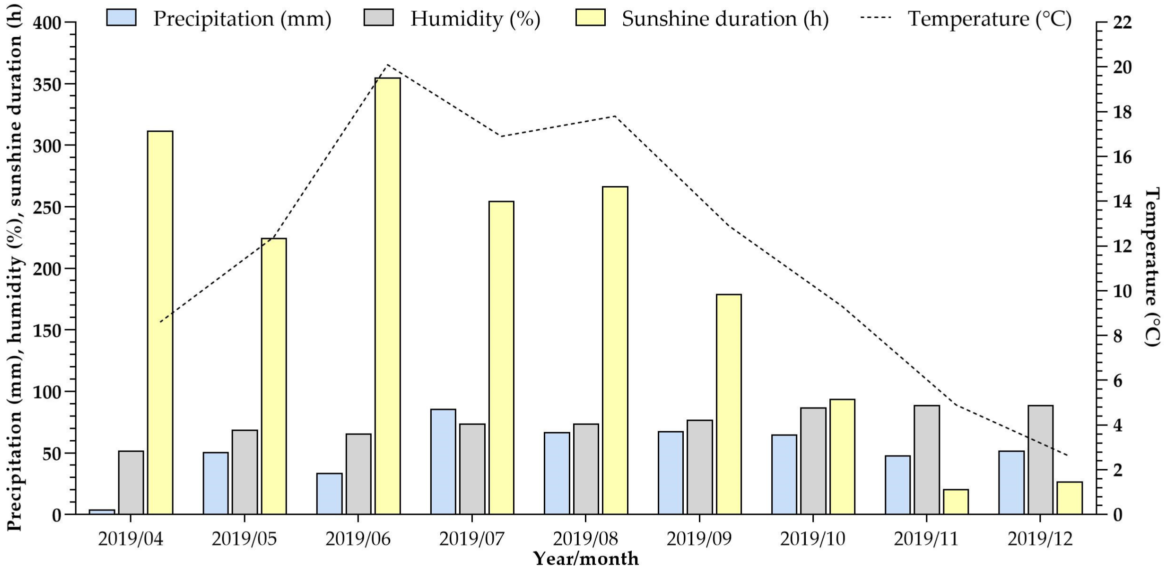

Figure S1. Climatic data in Lithuania during lingonberry vegetation in 2019.

Table S1. Contents of anthocyanins (μg/g DW ± SD) in lingonberries, collected at different locations. Values marked with * in the same column indicate the highest ($p < 0.05$) amounts in samples. ND—not detected, NQ—not quantified.

Location	Delphinidin-3-*O*-galactoside	Delphinidin-3-*O*-glucoside	Cyanidin-3-*O*-galactoside	Cyanidin-3-*O*-glucoside	Cyanidin-3-*O*-arabinoside	Petunidin-3-*O*-glucoside	Peonidin-3-*O*-glucoside	Malvidin-3-*O*-glucoside	Cyanidin
Apūniškis	14.9 ± 0.5	NQ	2606.6 ± 37.2	184.9 ± 2.5	293.1 ± 3.2	9.7 ± 0.1	26.6 ± 1.0	NQ	8.1 ± 0.4
Plunksnuočiai	8.1 ± 0.6	ND	2970.5 ± 48.4	290.0 ± 11.5	486.5 ± 10.6	8.9 ± 0.8	44.6 ± 1.4	NQ	4.3 ± 0.1
Šakarva	11.5 ± 0.7	ND	3877.1 ± 131.0	428.2 ± 18.6	581.2 ± 18.7	9.9 ± 0.4	45.9 ± 1.6	1.4 ± 0.1	21.5 ± 0.4
Andrioniškis	8.7 ± 0.0	NQ	3785.6 ± 63.8	439.0 ± 7.3	539.1 ± 11.1	11.7 ± 0.2	40.6 ± 2.0	NQ	7.3 ± 0.4
Jurgionys	23.9 ± 1.0	1.0 ± 0.3	3668.2 ± 27.4	345.0 ± 4.2	457.3 ± 3.7	13.3 ± 0.0	37.4 ± 1.4	ND	12.9 ± 0.2
Kernai	22.7 ± 0.0	0.7 ± 0.1	3856.3 ± 45.0	269.5 ± 9.7	579.1 ± 12.9	12.7 ± 0.3	44.3 ± 1.4	ND	10.2 ± 0.3
Žadeikiai	ND	ND	2820.6 ± 38.0	221.0 ± 3.3	272.8 ± 6.1	8.8 ± 0.3	24.2 ± 0.1	NQ	3.6 ± 0.1
Galvokai	ND	ND	1690.5 ± 68.1	113.3 ± 5.0	136.4 ± 3.6	NQ	16.3 ± 0.2	ND	2.9 ± 0.1
Giteniškė	14.4 ± 0.2	ND	4646.9 ± 107.8	299.1 ± 7.2	535.3 ± 11.3	12.3 ± 0.1	47.9 ± 1.6	NQ	44.2 ± 1.1*
Šalčininkėliai	NQ	ND	3470.9 ± 119.7	476.2 ± 13.2	786.4 ± 17.0	NQ	62.1 ± 4.5*	NQ	7.8 ± 0.3
Bitėnai	63.9 ± 0.4*	2.1 ± 0.6*	6252.1 ± 20.0*	663.3 ± 3.5*	597.3 ± 2.9	20.9 ± 0.1*	38.6 ± 0.5	3.4 ± 0.2	12.4 ± 0.2
Pagramantis	16.2 ± 0.7	ND	3531.2 ± 17.4	388.2 ± 2.5	624.9 ± 4.3	19.0 ± 0.6*	51.3 ± 0.8	1.1 ± 0.1	14.8 ± 0.5
Kūprė	29.3 ± 0.5	ND	2553.4 ± 50.0	274.1 ± 6.6	344.4 ± 22.3	NQ	38.7 ± 1.2	ND	38.9 ± 0.8
Bruknynė	NQ	NQ	3008.3 ± 175.7	417.0 ± 20.1	663.1 ± 29.0	9.3 ± 0.1	51.8 ± 1.5	0.4 ± 0.0	9.9 ± 0.2
Viršilai	12.3 ± 0.1	NQ	2537.9 ± 49.2	184.6 ± 3.4	316.6 ± 14.3	9.9 ± 0.3	26.7 ± 0.3	ND	10.7 ± 0.3
Labanoras (a)	ND	ND	221.7 ± 5.4	10.4 ± 0.2	3.9 ± 0.1	ND	ND	ND	ND
Labanoras (b)	ND	NQ	2449.1 ± 91.7	244.7 ± 17.3	351.4 ± 14.3	10.6 ± 0.2	28.8 ± 0.8	ND	9.5 ± 0.3
Komarinė	13.5 ± 0.3	2.3 ± 1.1*	3013.3 ± 128.2	306.9 ± 11.8	506.3 ± 20.5	14.1 ± 0.6	43.1 ± 1.2	6.7 ± 0.3*	13.6 ± 0.2
Marcinkonys	NQ	ND	3221.6 ± 149.3	394.5 ± 19.6	434.7 ± 22.7	10.2 ± 0.0	41.9 ± 0.4	1.3 ± 0.1	18.2 ± 0.5
Šilainė	ND	ND	2546.7 ± 43.5	171.6 ± 6.5	288.9 ± 4.8	NQ	33.1 ± 1.6	ND	29.1 ± 0.2
Smėlynė	9.3 ± 0.0	ND	3350.7 ± 43.6	296.4 ± 3.8	292.1 ± 3.0	9.7 ± 0.3	28.1 ± 1.1	ND	16.3 ± 0.1
Ilgalaukiai	15.4 ± 0.2	ND	2793.4 ± 124.0	329.1 ± 14.8	563.0 ± 22.6	9.1 ± 0.1	45.7 ± 2.6	NQ	7.1 ± 0.4
Vosniūnai	NQ	ND	4135.6 ± 188.1	350.0 ± 8.5	501.1 ± 25.5	9.9 ± 0.1	42.5 ± 2.1	NQ	15.4 ± 0.1
Kukuliškiai	10.6 ± 0.0	ND	3498.7 ± 81.2	333.5 ± 9.2	609.5 ± 20.1	11.5 ± 0.2	47.7 ± 2.0	NQ	13.1 ± 0.1
Tolkūnai	ND	ND	3271.9 ± 5.6	244.8 ± 1.6	523.8 ± 1.0	9.7 ± 0.2	68.2 ± 0.7*	0.5 ± 0.0	4.6 ± 0.3
Bakūriškis	8.3 ± 0.0	NQ	2684.0 ± 3.2	182.4 ± 0.7	220.4 ± 1.7	11.9 ± 0.6	24.2 ± 1.0	ND	8.6 ± 0.4
Šilinė	NQ	ND	3065.0 ± 68.5	410.4 ± 6.5	627.1 ± 8.8	10.3 ± 0.6	40.8 ± 1.1	ND	14.2 ± 0.3
Tyrelis	8.0 ± 0.3	ND	3215.8 ± 19.0	332.6 ± 1.5	438.8 ± 3.7	10.1 ± 0.5	40.4 ± 0.5	NQ	20.3 ± 0.7
Pažemys	8.5 ± 0.3	ND	3969.6 ± 84.6	490.6 ± 78.9	864.3 ± 39.3*	10.8 ± 0.5	68.2 ± 3.7*	1.9 ± 0.0	25.4 ± 0.6

Table S2. Contents of flavan-3-ols and proanthocyanidins (μg/g DW ± SD) in lingonberries, collected at different locations. Values marked with * in the same column indicate the highest ($p < 0.05$) amounts in samples.

Location	(+)-Catechin	(−)-Epicatechin	Procyanidin A1	Procyanidin A2	Procyanidin A4	Procyanidin B1	Procyanidin B2	Procyanidin B3	Procyanidin C1
Apūniškis	3215.5 ± 98.9	105.6 ± 3.8	744.5 ± 12.2	203.9 ± 2.2	10.5 ± 0.3	1164.3 ± 38.2	323.2 ± 16.2	1248.5 ± 41	324.1 ± 15.0
Plunksnuočiai	2655.4 ± 34.6	128.9 ± 4.5	642.4 ± 8.1	392.7 ± 3.2	13.2 ± 1.2	1033.9 ± 17.1	359.0 ± 28.9	1155.8 ± 41.5	369.7 ± 10.9
Šakarva	2363.6 ± 9.8	77.8 ± 4.8	731.5 ± 23.4	247.3 ± 0.9	6.9 ± 0.7	1018.9 ± 28.8	460.5 ± 23.7	1239.7 ± 31.6	362.6 ± 13.0
Andrioniškis	3650.5 ± 111.9	130.4 ± 2.8	748.6 ± 13.5	293.3 ± 11.9	17.9 ± 0.8	1475.5 ± 7.5	584.7 ± 23.4	1400.0 ± 18.2	474.5 ± 8.1
Jurgionys	3045.4 ± 61.7	89.1 ± 1.7	834.0 ± 2.3	270.0 ± 6.5	6.9 ± 0.2	1458.7 ± 9.3	611.9 ± 4.8	1323.7 ± 27.3	303.8 ± 9.7
Kernai	2196.3 ± 9.9	80.2 ± 5.6	545.9 ± 6.2	471.7 ± 3.4	4.8 ± 0.8	715.3 ± 36.7	345.8 ± 3.3	953.9 ± 25.5	452.9 ± 4.4
Žadeikiai	2982.8 ± 17.6	93.1 ± 0.5	718.6 ± 38.6	245.6 ± 10.5	9.7 ± 0.6	1283.5 ± 49.5	346.4 ± 3.4	1245.8 ± 78.8	342.9 ± 5.2
Galvokai	2036.6 ± 48.1	37.4 ± 3.2	387.5 ± 15.1	148.9 ± 7.4	6.2 ± 0.5	783.2 ± 27.6	372.9 ± 7.3	895.3 ± 52.9	214.4 ± 9.2
Giteniškė	3345.0 ± 63.4	77.5 ± 1.5	924.1 ± 29.5	560.9 ± 23.2	14.2 ± 4.1	1096.6 ± 41.2	433.0 ± 1.8	1305.2 ± 14.7	522.7 ± 18.2
Šalčininkėliai	3824.7 ± 145.3	156.9 ± 6.3	1050.0 ± 32.9	258.9 ± 6.3	19.4 ± 1.5	1256.7 ± 72.4	564.5 ± 35.7	1312.5 ± 60.5	307.1 ± 23.9
Bitėnai	4974.6 ± 102.9*	129.9 ± 5.5	1428.4 ± 17.9*	438.4 ± 0.8	46.2 ± 1.1*	1710.4 ± 33.5	821.7 ± 7.0*	1887.7 ± 53.4	436.6 ± 12.2
Pagramantis	2532.0 ± 144.1	82.5 ± 2.7	679.0 ± 12.7	339.2 ± 22.6	9.1 ± 0.4	980.7 ± 41.7	482.7 ± 19.2	990.2 ± 2.5	367.3 ± 7.3
Kūprė	3710.7 ± 203.8	95.0 ± 2.2	1076.1 ± 17.4	254.5 ± 9.2	7.0 ± 0.4	1493.6 ± 88.0	463.0 ± 16.4	1316.7 ± 30.5	404.8 ± 5.6
Bruknynė	3628.2 ± 178.9	145.0 ± 5.0	1010.8 ± 54.3	274.9 ± 20.2	21.1 ± 0.5	1233.6 ± 62.3	536.4 ± 6.1	1370.8 ± 41.4	297.4 ± 7.8
Viršilai	3367.8 ± 106.1	152.9 ± 9.1	877.3 ± 54.3	300.3 ± 11.9	17.0 ± 0.4	1387.6 ± 50.6	403.1 ± 20.0	1546.8 ± 31.0	477.1 ± 30.3
Labanoras (a)	2028.1 ± 69.5	174.9 ± 1.3	745.3 ± 29.9	294.4 ± 8.0	15.1 ± 0.3	881.0 ± 18.4	309.1 ± 2.8	915.0 ± 19.6	405.0 ± 10.1
Labanoras (b)	2810.0 ± 131.3	130.9 ± 1.1	667.5 ± 22.5	209.0 ± 7.4	9.5 ± 0.5	1035.0 ± 43.5	595.3 ± 10.6	1115.1 ± 40.6	390.6 ± 1.2
Komarinė	2901.9 ± 60.6	98.9 ± 2.3	756.5 ± 41.6	393.8 ± 23.6	13.0 ± 0.7	1165.4 ± 37.9	570.7 ± 15.2	1191.6 ± 34.5	470.0 ± 25.1
Marcinkonys	5305.0 ± 108.8*	152.1 ± 1.8	1306.8 ± 43.0*	417.4 ± 9.5	26.3 ± 2.0	2561.3 ± 8.0*	639.9 ± 3.6	2306.4 ± 79.5*	792.4 ± 14.2*
Šilainė	3708.4 ± 162.9	115.3 ± 3.9	854.9 ± 42.4	238.0 ± 4.8	18.1 ± 1.2	1705.7 ± 71.2	405.1 ± 3.9	1415.1 ± 70.7	512.3 ± 13.3
Smėlynė	3333.3 ± 69.7	146.4 ± 8.5	728.5 ± 31.0	252.3 ± 8.4	15.6 ± 0.5	1448.4 ± 34.2	509.9 ± 28.8	1286.1 ± 63.2	493.2 ± 8.0
Ilgalaukiai	2593.3 ± 43.8	75.6 ± 5.1	757.5 ± 0.3	425.4 ± 7.2	12.1 ± 0.1	1181.8 ± 17.2	314.4 ± 10.0	1074.7 ± 7.2	482.6 ± 12.7
Vosniūnai	3612.1 ± 52.1	173.8 ± 7.5	749.3 ± 36.1	258.6 ± 23.5	10.9 ± 0.2	1157.2 ± 54.2	644.6 ± 26.8	1213.6 ± 46.4	411.3 ± 24.1
Kukuliškiai	2767.3 ± 135.3	103.9 ± 2.2	747.9 ± 39.7	396.8 ± 4.1	11.3 ± 0.1	1173.3 ± 18.1	472.2 ± 16.8	1257.4 ± 40.7	486.4 ± 8.7
Tolkūnai	4820.0 ± 78.8	214.5 ± 8.2*	1285.3 ± 39.6	428.6 ± 15.0	24.1 ± 1.4	2399.0 ± 82.2*	343.4 ± 4.7	2082.3 ± 61.5	790.2 ± 36.1*
Bakūriškis	1678.0 ± 112.7	57.1 ± 0.1	442.0 ± 34.4	618.3 ± 2.0	15.8 ± 1.2	596.4 ± 14.3	268.5 ± 14.2	701.6 ± 25.5	768.8 ± 2.9*
Šilinė	2947.5 ± 56.0	101.8 ± 4.6	649.0 ± 14.5	348.2 ± 29.8	15.9 ± 1.1	1164.1 ± 55.1	366.8 ± 20.5	1240.1 ± 50.6	474.2 ± 32.5
Tyrelis	3234.4 ± 40.3	96.4 ± 0.3	771.0 ± 3.7	272.9 ± 1.8	13.4 ± 0.8	1469.0 ± 23.8	354.0 ± 6.3	1292.2 ± 27.8	459.0 ± 2.7
Pažemys	2081.9 ± 120.3	16.7 ± 0.6	704.7 ± 43.7	909.9 ± 30.9*	24.1 ± 1.1	725.3 ± 14.8	339.1 ± 2.2	1143.3 ± 41.4	632.7 ± 17.8

Table S3. Contents of flavonol glycosides (μg/g DW ± SD) in lingonberries, collected at different locations. Values marked with * in the same column indicate the highest ($p < 0.05$) amounts in samples. ND—not detected.

Location	Rutin	Hyperoside	Isoquercitrin	Reynoutrin	Guaiaverin	Avicularin	Quercitrin	Quercetin-HMG-rhamnoside	Astragalin	Afzelin
Apūniškis	ND	70.6 ± 0.7	45.4 ± 0.6	29.5 ± 0.4	13.1 ± 0.1	93.9 ± 1.0	254.2 ± 2.8	141.1 ± 0.9	4.3 ± 0.4	7.9 ± 0.4
Plunksnuočiai	7.3 ± 0.2*	99.9 ± 4.7	57.5 ± 3.5	41.1 ± 1.3	16.2 ± 1.3	135.2 ± 4.1	278.8 ± 5.5	152.5 ± 4.3	2.7 ± 0.3	3.9 ± 0.2
Šakarva	ND	54.8 ± 1.3	26.6 ± 0.0	46.2 ± 1.2	12.8 ± 0.0	114.7 ± 2.4	203.8 ± 5.3	122.0 ± 2.0	ND	2.6 ± 0.1
Andrioniškis	ND	74.5 ± 0.5	53.0 ± 0.1	51.8 ± 0.3	13.3 ± 0.1	120.1 ± 0.8	345.2 ± 1.3	171.0 ± 1.7	ND	10.5 ± 0.3
Jurgionys	ND	95.4 ± 1.7	51.8 ± 0.3	60.6 ± 0.5	25.1 ± 0.3	158.7 ± 2.0	139.0 ± 0.7	90.6 ± 0.8	ND	2.1 ± 0.1
Kernai	ND	71.8 ± 0.6	36.5 ± 0.2	40.7 ± 0.2	11.8 ± 0.2	109.7 ± 0.5	198.3 ± 0.8	85.8 ± 0.8	ND	1.9 ± 0.4
Žadeikiai	ND	132.2 ± 4.6	64.6 ± 3.7	51.3 ± 0.7	22.8 ± 0.8	171.1 ± 10	329.6 ± 16.6	203.8 ± 5.7	ND	2.3 ± 0.1
Galvokai	ND	296.9 ± 1.1*	133.3 ± 4.2*	77.0 ± 3.7	48.6 ± 2.7	320.1 ± 7.9*	410.5 ± 9.9	181.7 ± 7.8	ND	7.5 ± 0.8
Giteniškė	ND	65.5 ± 2.9	31.7 ± 2.0	45.0 ± 1.0	14.8 ± 0.5	128.9 ± 0.4	278.4 ± 4.3	169.5 ± 3.0	ND	4.3 ± 0.2
Šalčininkėliai	ND	113.1 ± 2.3	52.1 ± 3.4	56.9 ± 2.5	17.2 ± 0.8	174.6 ± 8.7	323.0 ± 13.6	243.4 ± 7.6	ND	6.4 ± 0.5
Bitėnai	ND	73.2 ± 0.9	35.9 ± 1.3	92.3 ± 3.0	22.9 ± 0.3	139.8 ± 0.2	289.3 ± 1.7	243.5 ± 1.0	ND	9.3 ± 0.4
Pagramantis	3.1± 0.0	93.6 ± 2.6	43.1 ± 2.5	64.5 ± 4.4	23.8 ± 1.2	167.5 ± 9.7	349.2 ± 15.9	171.1 ± 8.7	ND	7.2 ± 0.4
Kūprė	ND	113.9 ± 5.6	54.4 ± 2.2	53.4 ± 3.9	23.2 ± 0.9	157.9 ± 5.0	244.8 ± 8.1	117.4 ± 2.3	ND	6.8 ± 0.6
Bruknynė	ND	114.5 ± 4.3	52.6 ± 3.8	56.8 ± 3.1	17.9 ± 1.2	171.3 ± 9.2	317.5 ± 7.1	241.1 ± 15.4	ND	6.1 ± 0.0
Viršilai	ND	95.3 ± 4.5	57.4 ± 1.8	42.3 ± 3.1	18.6 ± 0.7	130.2 ± 2.8	381.2 ± 14.2	209.3 ± 9.1	3.8 ± 0.3	9.3 ± 0.6
Labanoras (a)	ND	119.9 ± 3.3	60.7 ± 1.7	79.1 ± 2.4	26.7 ± 0.5	270.5 ± 9.9	562.7 ± 15.9*	17.3 ± 0.0	ND	17.2 ± 0.6*
Labanoras (b)	ND	179.5 ± 6.6	74.8 ± 1.8	65.5 ± 1.5	26.7 ± 0.6	206.5 ± 5.5	89.1 ± 2.3	83.4 ± 2.2	ND	0.4 ± 0.1
Komarinė	3.3 ± 0.1	129.9 ± 1.7	58.7 ± 2.0	76.9 ± 2.0	30.7 ± 2.2	206.9 ± 6.3	413.7 ± 7.6	185.1 ± 5.4	3.4 ± 0.1	7.7 ± 0.4
Marcinkonys	ND	149.4 ± 4.6	79.5 ± 1.9	76.1 ± 1.2	32.8 ± 1.4	180.1 ± 5.4	489.7 ± 8.8	233.0 ± 4.1	5.0 ± 0.4	16.6 ± 0.7*
Šilainė	ND	120.3 ± 6.6	61.4 ± 7.0	41.9 ± 1.5	20.2 ± 0.7	150.4 ± 18.1	104.5 ± 2.2	103.3 ± 2.7	ND	1.9 ± 0.0
Smėlynė	ND	300.6 ± 14.7*	120.3 ± 8.8*	83.2 ± 2.8	31.7 ± 2.7	253.7 ± 6.2	272.3 ± 7.3	74.6 ± 4.6	ND	3.0 ± 0.2
Ilgalaukiai	ND	73.9 ± 6.0	36.9 ± 2.8	46.7 ± 1.9	15.5 ± 0.9	128.2 ± 5.2	272.3 ± 4.9	90.8 ± 1.2	2.8 ± 0.1	6.0 ± 0.4
Vosniūnai	3.0 ± 0.1	174.5 ± 3.0	94.4 ± 1.0	74.1 ± 2.9	39.3 ± 0.5	206.9 ± 2.9	590.7 ± 21.9*	234.3 ± 2.0	4.9 ± 0.1	13.3 ± 0.5
Kukuliškiai	3.7 ± 0.2	125.4 ± 8.4	56.5 ± 4.1	79.9 ± 4.2	31.5 ± 2.4	209.5 ± 6.6	430.4 ± 3.6	176.6 ± 9.2	5.4 ± 0.1*	10.6 ± 0.6
Tolkūnai	ND	137.6 ± 6.4	91.3 ± 3.0	106.2 ± 2.1*	46.4 ± 1.6	281.7 ± 7.6	514.3 ± 12.9	326.4 ± 10.8*	ND	7.0 ± 0.4
Bakūriškis	ND	63.8 ± 2.1	52.4 ± 0.7	35.6 ± 1.2	12.3 ± 0.6	106.8 ± 5.1	433.6 ± 5.1	272.5 ± 2.1	3.2 ± 0.0	8.4 ± 0.0
Šilinė	ND	116.6 ± 5.3	56.9 ± 2.0	39.0 ± 0.8	21.3 ± 0.3	143.5 ± 8.2	421.1 ± 21.7	141.2 ± 8.9	3.1 ± 0.0	7.7 ± 0.2
Tyrelis	ND	106.8 ± 0.3	54.3 ± 0.6	50.0 ± 0.6	21.7 ± 0.4	137.4 ± 1.1	294.2 ± 5.5	145.9 ± 2.3	3.8 ± 0.1	7.8 ± 0.1
Pažemys	ND	119.3 ± 6.1	68.0 ± 4.7	50.7 ± 1.0	15.8 ± 1.0	161.1 ± 7.8	47.9 ± 0.3	59.1 ± 1.5	ND	0.3 ± 0.0

Table S4. Contents of phenolic acids (μg/g DW ± SD) in lingonberries, collected at different locations. Values marked with * in the same column indicate the highest ($p < 0.05$) amounts in samples. ND—not detected, NQ—not quantified.

Location	Chlorogenic acid	Cryptochlorogenic acid	Neochlorogenic acid	*p*-Coumaric acid	Sinapic acid	Ferulic acid	Vanillic acid	Protocatechuic acid
Apūniškis	115.7 ± 6.7	67.6 ± 0.6	28.0 ± 0.4	37.8 ± 0.9	1.4 ± 0.1	9.6 ± 0.2	16.1 ± 0.2	69.5 ± 1.9
Plunksnuočiai	112.0 ± 5.3	85.6 ± 0.6	27.5 ± 1.8	56.8 ± 0.3	NQ	7.0 ± 0.5	12.2 ± 0.8	62.2 ± 3.0
Šakarva	300.7 ± 8.5	89.9 ± 0.7	22.2 ± 0.3	44.9 ± 1.1	7.5 ± 0.6	15.4 ± 0.6	13.6 ± 0.8	73.0 ± 2.1
Andrioniškis	219.0 ± 11.4	111.8 ± 1.6	28.1 ± 0.1	33.8 ± 0.4	3.3 ± 0.0	10.2 ± 0.1	21.8 ± 0.2	94.9 ± 0.4
Jurgionys	155.8 ± 2.5	94.7 ± 0.6	22.5 ± 0.8	25.7 ± 0.2	1.8 ± 0.1	9.1 ± 0.0	11.8 ± 0.7	143.9 ± 1.2*
Kernai	114.8 ± 3.1	91.4 ± 3.2	23.9 ± 0.3	59.4 ± 0.6	4.0 ± 0.0	12.6 ± 0.6	11.0 ± 0.7	68.8 ± 3.0
Žadeikiai	136.9 ± 6.7	94.3 ± 1.4	23.3 ± 1.6	19.1 ± 1.4	0.7 ± 0.0	7.0 ± 0.5	11.8 ± 0.5	80.7 ± 2.4
Galvokai	92.2 ± 4.6	79.0 ± 1.4	40.6 ± 2.6*	14.3 ± 0.7	0.3 ± 0.1	6.1 ± 0.2	10.0 ± 0.8	62.2 ± 1.9
Giteniškė	111.1 ± 1.5	127.9 ± 4.1	20.9 ± 0.1	31.2 ± 1.2	2.6 ± 0.1	10.4 ± 0.1	22.8 ± 1.3	76.5 ± 2.8
Šalčininkėliai	169.2 ± 6.4	136.2 ± 4.2	25.5 ± 3.2	81.7 ± 2.8	NQ	4.5 ± 0.3	7.4 ± 0.1	77.5 ± 2.5
Bitėnai	470.3 ± 1.4*	188.4 ± 1.9*	41.1 ± 0.4*	72.8 ± 0.6	5.3 ± 0.3	12.5 ± 0.7	34.1 ± 1.3*	122.8 ± 2.8
Pagramantis	204.1 ± 7.1	104.7 ± 4.4	22.6 ± 2.1	48.7 ± 0.2	3.9 ± 0.1	11.4 ± 0.5	23.2 ± 1.9	98.0 ± 3.1
Kūprė	277.9 ± 9.6	123.8 ± 5.8	18.1 ± 0.8	17.7 ± 1.4	NQ	5.0 ± 0.4	19.2 ± 1.2	82.4 ± 5.8
Bruknynė	169.0 ± 7.8	136.7 ± 4.7	26.2 ± 1.8	86.3 ± 1.5	NQ	4.9 ± 0.1	9.3 ± 0.5	83.4 ± 4.1
Viršilai	200.2 ± 4.0	100.9 ± 5.1	30.8 ± 0.7	71.4 ± 0.9	2.1 ± 0.2	10.0 ± 0.5	18.4 ± 0.8	94.2 ± 5.9
Labanoras (a)	332.0 ± 9.4	ND	32.0 ± 0.5	44.9 ± 1.1	10.3 ± 0.1	17.5 ± 0.0	18.8 ± 0.0	68.2 ± 1.6
Labanoras (b)	299.2 ± 7.7	121.6 ± 5.3	21.0 ± 0.6	56.0 ± 0.3	1.3 ± 0.0	9.1 ± 0.2	20.2 ± 0.4	80.9 ± 4.1
Komarinė	209.5 ± 5.8	143.7 ± 7.6	23.1 ± 1.5	54.0 ± 2.5	7.1 ± 0.5	13.8 ± 0.7	25.3 ± 1.1	102.4 ± 5.3
Marcinkonys	368.4 ± 6.5	147.7 ± 7.4	32.6 ± 0.0	107.5 ± 4.9*	NQ	5.9 ± 0.2	38.6 ± 2.3*	152.4 ± 0.1*
Šilainė	456.5 ± 8.5*	114.0 ± 3.1	14.7 ± 0.9	67.8 ± 2.7	NQ	4.6 ± 0.2	25.2 ± 2.4	98.9 ± 4.5
Smėlynė	323.7 ± 12.5	135.7 ± 5.0	21.7 ± 0.0	23.3 ± 1.6	NQ	4.4 ± 0.5	17.1 ± 1.0	81.3 ± 0.2
Ilgalaukiai	98.2 ± 2.5	108.4 ± 1.1	22.1 ± 0.5	43.1 ± 0.1	5.6 ± 0.1	12.8 ± 0.1	18.8 ± 0.8	72.3 ± 0.7
Vosniūnai	202.9 ± 11.1	175.5 ± 3.7*	28.0 ± 1.3	38.0 ± 3.3	4.8 ± 0.4	12.0 ± 0.7	22.9 ± 1.0	78.8 ± 3.6
Kukuliškiai	194.7 ± 8.6	154.6 ± 0.5	22.5 ± 0.5	45.4 ± 0.4	8.3 ± 0.5	15.1 ± 1.1	27.5 ± 1.2	110.5 ± 4.8
Tolkūnai	181.8 ± 1.7	135.9 ± 2.4	24.9 ± 0.7	92.5 ± 0.5	33.6 ± 0.5*	39.3 ± 0.6*	36.7 ± 1.6*	122.4 ± 4.2
Bakūriškis	47.3 ± 4.0	130.0 ± 1.4	21.3 ± 0.1	60.1 ± 0.9	NQ	6.2 ± 0.6	23.5 ± 0.9	60.9 ± 2.3
Šilinė	188.6 ± 9.9	149.8 ± 6.4	25.1 ± 1.4	54.8 ± 0.4	4.0 ± 0.1	11.8 ± 0.6	21.0 ± 1.2	85.2 ± 4.6
Tyrelis	269.1 ± 4.2	132.9 ± 1.4	21.8 ± 0.1	58.2 ± 0.5	NQ	7.0 ± 0.1	21.1 ± 0.1	91.6 ± 0.9
Pažemys	122.0 ± 3.4	188.2 ± 9.0*	37.9 ± 2.2*	44.8 ± 1.9	3.2 ± 0.2	11.7 ± 0.4	17.3 ± 1.0	62.4 ± 4.5

Table S5. Contents of simple phenolics, phenolic acids precursors, flavonol aglycones, and stilbenes (μg/g DW ± SD) in lingonberries, collected at different locations. Values marked with * in the same column indicate the highest (p < 0.05) amounts in samples. ND—not detected, NQ—not quantified.

Location	Arbutin	2-*O*-Caffeoylarbutin	Benzoic acid	*trans*-Cinnamic acid	Quercetin	Kaempferol	Resveratrol
Apūniškis	297.3 ± 5.6	21.3 ± 0.2	1053.2 ± 12.5	11.0 ± 0.1	30.8 ± 0.2	1.93 ± 0.1	NQ
Plunksnuočiai	315.6 ± 20.2	16.4 ± 0.2	602.5 ± 31.3	18.2 ± 1.1	30.1 ± 0.3	NQ	NQ
Šakarva	289.5 ± 12.1	34.7 ± 1.0	1353.1 ± 52.0	10.2 ± 0.1	28.5 ± 0.1	NQ	ND
Andrioniškis	452.4 ± 3.3	26.3 ± 0.2	971.1 ± 2.8	20.9 ± 0.1	35.1 ± 0.5	ND	ND
Jurgionys	321.2 ± 6.4	22.1 ± 0.1	1160.5 ± 0.8	10.5 ± 0.2	35.9 ± 0.4	NQ	NQ
Kernai	345.7 ± 14.9	20.2 ± 0.1	1109.0 ± 17.5	18.8 ± 0.1	32.1 ± 0.1	ND	NQ
Žadeikiai	357.4 ± 16.7	26.5 ± 1.9	738.9 ± 39.3	10.5 ± 0.4	32.0 ± 0.7	NQ	ND
Galvokai	375.1 ± 18.1	25.5 ± 1.1	376.1 ± 11.9	6.7 ± 0.3	38.0 ± 1.0	ND	ND
Giteniškė	520.3 ± 22.3	34.7 ± 1.5	1087.4 ± 37.0	10.1 ± 0.2	33.8 ± 0.2	ND	NQ
Šalčininkėliai	542.3 ± 20.7	26.9 ± 1.3	570.4 ± 34.2	11.2 ± 0.6	29.5 ± 0.7	ND	ND
Bitėnai	513.8 ± 8.3	70.3 ± 0.9*	4759.3 ± 59.1*	47.9 ± 0.8*	47.1 ± 0.1*	NQ	1.7 ± 0.1*
Pagramantis	441.6 ± 23.1	23.5 ± 1.7	1833.5 ± 61.9	21.0 ± 1.1	47.0 ± 0.7*	ND	NQ
Kūprė	460.1 ± 12.0	27.7 ± 0.8	377.2 ± 6.2	8.9 ± 0.3	28.6 ± 0.3	ND	ND
Bruknynė	567.1 ± 29.7	29.4 ± 0.9	588.0 ± 16.9	12.7 ± 0.1	35.1 ± 0.5	ND	ND
Viršilai	469.5 ± 32.9	31.1 ± 0.1	1264.4 ± 50.7	14.9 ± 1.1	35.9 ± 1.9	NQ	NQ
Labanoras (a)	349.4 ± 7.0	NQ	1877.5 ± 51	13.3 ± 0.1	37.4 ± 0.4	ND	NQ
Labanoras (b)	342.4 ± 18.8	33.7 ± 2.0	743.2 ± 1.1	19.8 ± 0.7	40.5 ± 1.4	ND	NQ
Komarinė	469.9 ± 25.7	26.6 ± 1.3	2364.4 ± 77.9	25.6 ± 1.9	45.6 ± 3.1	2.34 ± 0.1*	1.3 ± 0.0
Marcinkonys	405.6 ± 3.3	29.2 ± 0.3	816.0 ± 4.7	30.4 ± 0.2	33.3 ± 0.4	1.92 ± 0.0	NQ
Šilainė	425.4 ± 22.6	17.0 ± 1.0	319.6 ± 22.2	6.9 ± 0.4	31.3 ± 0.8	NQ	NQ
Smėlynė	356.2 ± 17.0	16.1 ± 0.7	417.0 ± 15.8	9.1 ± 0.1	32.2 ± 0.5	ND	NQ
Ilgalaukiai	317.1 ± 4.2	23.5 ± 0.1	1596 ± 22.1	11.9 ± 0.2	30.6 ± 0.2	NQ	NQ
Vosniūnai	449.5 ± 17.2	21.7 ± 0.5	1657.2 ± 34.9	24.2 ± 0.7	38.5 ± 1.8	2.5 ± 0.1*	NQ
Kukuliškiai	433.9 ± 22.3	25.7 ± 1.6	2461.9 ± 66.4	25.5 ± 0.4	46.8 ± 1.1*	2.0 ± 0.0	1.4 ± 0.1
Tolkūnai	681.4 ± 21.6*	41.1 ± 0.9	444.9 ± 27.1	7.8 ± 0.2	37.4 ± 0.5	NQ	NQ
Bakūriškis	512.4 ± 8.8	28.8 ± 0.1	875.8 ± 0.8	17.4 ± 0.1	41.8 ± 0.5	ND	NQ
Šilinė	370.4 ± 16.8	31.3 ± 0.2	1046.9 ± 25.2	11.6 ± 0.6	34.0 ± 1.5	ND	NQ
Tyrelis	299.8 ± 4.0	21.2 ± 0.7	811.2 ± 12.1	17.3 ± 0.4	32.8 ± 0.1	NQ	NQ
Pažemys	360.0 ± 11.0	39.4 ± 1.4	812.9 ± 39.2	20.6 ± 0.8	31.8 ± 0.5	ND	NQ

Table S6. Contents of triterpenoid acids and sterols (μg/g DW ± SD) in lingonberries, collected at different locations. Values marked with * in the same column indicate the highest ($p < 0.05$) amounts in samples. ND—not detected, NQ—not quantified.

Location	Maslinic acid	Corosolic acid	Betulinic acid	Oleanolic acid	Ursolic acid	β-Sitosterol
Apūniškis	18.8 ± 0.2	38.0 ± 1.8	4.2 ± 0.2	726.4 ± 7.4	4306.7 ± 18.2	774.9 ± 3.7
Plunksnuočiai	9.3 ± 0.5	26.3 ± 1.4	NQ	692.6 ± 25.2	3966.5 ± 166.2	795.8 ± 26.3
Šakarva	15.8 ± 0.7	34.0 ± 2.2	6.3 ± 0.1	714.8 ± 34.5	3772.8 ± 84.8	779.0 ± 17.6
Andrioniškis	17.3 ± 0.3	32.7 ± 0.3	5.0 ± 0.2	829.1 ± 2.6	4630.2 ± 28.8	780.2 ± 8.7
Jurgionys	23.5 ± 0.1	49.0 ± 2.0*	11.7 ± 0.6*	1123.3 ± 7.6	4251.4 ± 115.9	716.9 ± 6.1
Kernai	21.8 ± 2.6	48.7 ± 0.2*	NQ	713.8 ± 28.3	4021.5 ± 80.8	750.3 ± 0.6
Žadeikiai	17.3 ± 0.4	30.6 ± 1.5	ND	799.3 ± 13.9	4499.6 ± 174.2	708.2 ± 2.8
Galvokai	21.7 ± 0.5	47.2 ± 0.8*	ND	1047.8 ± 51.7	5777.5 ± 212.4*	773.8 ± 29.4
Giteniškė	12.6 ± 0.7	20.4 ± 1.2	6.6 ± 0.1	614.7 ± 22.6	3374.2 ± 175.2	771.6 ± 6.5
Šalčininkėliai	10.3 ± 0.1	24.9 ± 0.7	NQ	664.4 ± 17.5	3507.6 ± 193.1	618.8 ± 23.7
Bitėnai	12.2 ± 0.3	14.1 ± 0.7	NQ	450.9 ± 2.4	2355.9 ± 10.3	1028.2 ± 2.6*
Pagramantis	24.1 ± 0.1	29.8 ± 0.2	7.1 ± 0.5	890.5 ± 13.1	4706.4 ± 44.2	695.9 ± 2.9
Kūprė	11.6 ± 0.8	10.0 ± 0.4	ND	489.1 ± 25.3	2640.5 ± 70.6	613.8 ± 12.9
Bruknynė	8.0 ± 0.6	20.7 ± 0.6	NQ	558.1 ± 33.6	2862.6 ± 71.0	620.1 ± 17.5
Viršilai	15.4 ± 0.4	23.9 ± 0.7	NQ	633.7 ± 10.4	3304.5 ± 104	870.4 ± 44.0
Labanoras (a)	24.7 ± 0.8	41.9 ± 2.6	2.5 ± 0.1	1398.5 ± 57.1*	3658.0 ± 122.0	953.4 ± 14.1
Labanoras (b)	9.6 ± 0.2	16.8 ± 0.3	NQ	394.7 ± 138.8	1641.5 ± 154.9	656.0 ± 31.0
Komarinė	11.3 ± 0.4	23.3 ± 0.9	5.0 ± 0.5	542 ± 18.1	2582.9 ± 110.9	655.0 ± 0.5
Marcinkonys	29.9 ± 1.3	42.3 ± 2.0	3.6 ± 0.3	763 ± 42.8	3737.6 ± 164.2	935.0 ± 18.4
Šilainė	9.7 ± 0.5	27.6 ± 1.0	5.0 ± 0.4	885.6 ± 36.3	4891.9 ± 190.0	803.1 ± 36.0
Smėlynė	17.4 ± 0.9	51.0 ± 0.1*	2.8 ± 0.2	629.8 ± 0.8	3250.0 ± 42.5	826.0 ± 45.9
Ilgalaukiai	17.0 ± 1.5	28.4 ± 1.9	NQ	710.8 ± 31.6	3866.1 ± 77.8	754.2 ± 15.1
Vosniūnai	13.6 ± 0.0	18.7 ± 1.4	NQ	557.7 ± 12.4	2843.3 ± 168.6	835.5 ± 29.0
Kukuliškiai	17.3 ± 0.9	32.7 ± 0.3	7.0 ± 0.5	691.8 ± 8.4	3489.9 ± 76.1	758.8 ± 34.9
Tolkūnai	34.4 ± 1.6*	42.1 ± 0.6	ND	851.9 ± 8.9	4250.7 ± 97.6	901.2 ± 14.2
Bakūriškis	22.0 ± 1.5	42.0 ± 0.1	ND	715.3 ± 8.3	3996.2 ± 13.4	1055.1 ± 22.8*
Šilinė	19.0 ± 0.2	42.3 ± 1.1	2.8 ± 0.0	757.3 ± 13.6	4281.7 ± 152.4	789.9 ± 32.9
Tyrelis	24.0 ± 0.6	41.0 ± 1.1	3.1 ± 0.3	877.3 ± 0.8	4989.3 ± 9.8	859.6 ± 14.0
Pažemys	9.3 ± 0.5	23.9 ± 0.5	11.1 ± 0.7*	1004 ± 21.4	5285.8 ± 74.2*	849.4 ± 31.3

Table S7. Contents of neutral triterpenoids (μg/g DW ± SD) in lingonberries, collected at different locations. Values marked with * in the same column indicate the highest ($p < 0.05$) amounts in samples.

Location	Betulin	Erythrodiol	Uvaol	Lupeol	α-Amyrin	β-Amyrin	Friedelin
Apūniškis	240.5 ± 5.0	10.0 ± 0.8	54.8 ± 1.6	189.9 ± 2.5	490.1 ± 7.6	136.0 ± 9.0	389.2 ± 16.5
Plunksnuočiai	134.8 ± 7.0	6.7 ± 0.8	53.8 ± 0.5	235.9 ± 7.7	496.6 ± 17.2	106.8 ± 3.6	368.3 ± 5.1
Šakarva	214.9 ± 6.3	9.8 ± 3.3	51.1 ± 2.4	271.1 ± 1.1	624.7 ± 3.5	169.1 ± 3.9	268.9 ± 8.6
Andrioniškis	270.2 ± 3.6	14.9 ± 0.6	71.9 ± 2.2*	354.4 ± 17.3	344.2 ± 3.8	90.4 ± 2.3	197.2 ± 5.1
Jurgionys	266.2 ± 2.1	28.5 ± 1.9*	51.1 ± 2.4	311.5 ± 8.3	432.0 ± 5.6	136.3 ± 2.9	195.7 ± 7.9
Kernai	97.0 ± 4.6	15.5 ± 0.8	44.0 ± 1.2	301.6 ± 16.2	510.1 ± 8.7	171.6 ± 3.1	306.5 ± 15.1
Žadeikiai	151.9 ± 0.3	24.6 ± 0.1	51.4 ± 1.2	274.0 ± 16.1	486.5 ± 11.6	89.4 ± 0.4	237.7 ± 5.8
Galvokai	148.5 ± 3.6	30.6 ± 1.6*	77.4 ± 2.3*	315.3 ± 4.2	625.1 ± 6.2	141.4 ± 4.2	192.5 ± 8.3
Giteniškė	105.6 ± 5.7	3.3 ± 0.2	35.0 ± 0.2	240.2 ± 11.4	378.1 ± 18.8	64.3 ± 2.9	292.8 ± 12.9
Šalčininkėliai	115.5 ± 6.7	8.7 ± 0.5	56.1 ± 2.5	174.9 ± 0.3	441.7 ± 7.2	67.7 ± 4.0	285.6 ± 8.5
Bitėnai	131.9 ± 4.4	2.7 ± 0.4	22.2 ± 0.6	268.5 ± 0.4	275.0 ± 5.8	19.0 ± 1.3	91.5 ± 1.2
Pagramantis	180.3 ± 8.7	14.8 ± 1.1	50.7 ± 1.4	307.2 ± 20.5	573.1 ± 0.9	184.8 ± 13.8	455.1 ± 17.4*
Kūprė	54.3 ± 0.3	5.2 ± 0.0	25.3 ± 1.6	111.4 ± 4.0	325.4 ± 22.5	53.4 ± 4.4	94.5 ± 1.8
Bruknynė	97.9 ± 0.9	7.0 ± 1.0	42.4 ± 0.5	86.8 ± 0.8	425.3 ± 16.0	72.4 ± 1.7	203.1 ± 5.4
Viršilai	264.4 ± 3.2	11.0 ± 0.3	32.7 ± 1.0	115.7 ± 1.1	529.0 ± 10.2	112.5 ± 2.9	391.8 ± 11.2
Labanoras (a)	138.7 ± 7.2	28.5 ± 0.7*	72.1 ± 2.9*	859.1 ± 41.3*	577.7 ± 15.0	259.4 ± 10.6*	199.2 ± 9.4
Labanoras (b)	45.6 ± 1.6	2.5 ± 0.4	20.7 ± 0.2	106.2 ± 3.9	195.1 ± 10.3	79.6 ± 2.9	77.4 ± 0.6
Komarinė	165.2 ± 8.1	10.2 ± 0.3	28.1 ± 0.3	193.2 ± 8.1	514.4 ± 14.1	127.1 ± 0.4	359 ± 17.5
Marcinkonys	162.0 ± 8.0	14.2 ± 0.8	42.4 ± 0.1	180.8 ± 4.3	494.9 ± 21.7	113.0 ± 1.8	274.5 ± 2.3
Šilainė	200.5 ± 1.8	20.0 ± 1.4	74.1 ± 0.3*	362.2 ± 6.5	600.3 ± 1.9	111.2 ± 3.4	157.8 ± 1.1
Smėlynė	152.1 ± 1.9	6.3 ± 0.7	44.1 ± 1.2	371.7 ± 1.9	520.8 ± 1.5	63.0 ± 1.8	101.8 ± 7.6
Ilgalaukiai	214.7 ± 7.9	17.0 ± 0.5	38 ± 2.9	101.0 ± 6.2	694.1 ± 29.2	189.9 ± 5.7	432.8 ± 24.5
Vosniūnai	120.8 ± 4.9	20.2 ± 1.7	29.6 ± 0.4	166.5 ± 0.6	574.2 ± 30.1	79.9 ± 3.4	181.5 ± 4.8
Kukuliškiai	181.6 ± 9.3	26.2 ± 1.1	39.0 ± 2.1	212.7 ± 6.0	574.5 ± 31.0	142.0 ± 6.4	450.6 ± 15.8*
Tolkūnai	135.3 ± 3.1	11.3 ± 0.0	46.5 ± 2.4	165.8 ± 11.2	315.5 ± 0.9	66.1 ± 3.4	76.8 ± 2.1
Bakūriškis	152.6 ± 5.1	8.4 ± 0.8	41.7 ± 2.3	186.2 ± 3.6	468.8 ± 13.2	124.4 ± 5.2	230.0 ± 3.3
Šilinė	299.9 ± 3.9*	14.3 ± 0.4	46.9 ± 2.4	337.6 ± 4.1	666.5 ± 32.9	114.5 ± 3.1	373.8 ± 18.8
Tyrelis	190.2 ± 6.0	16.1 ± 0.1	70.4 ± 0.4*	260.3 ± 1.4	599.7 ± 18.6	143.5 ± 0.8	173.5 ± 1.8
Pažemys	319.8 ± 10.3*	14.0 ± 1.3	50.3 ± 3.0	343.6 ± 3.1	851.2 ± 19.9*	119.6 ± 7.7	485.8 ± 5.2*
